# A systematic review and meta-analysis of germline BRCA mutations in pancreatic cancer patients identifies global and racial disparities in access to genetic testing

**DOI:** 10.1016/j.esmoop.2023.100881

**Published:** 2023-02-21

**Authors:** S. Paiella, D. Azzolina, D. Gregori, G. Malleo, T. Golan, D.M. Simeone, M.B. Davis, P.G. Vacca, A. Crovetto, C. Bassi, R. Salvia, A.V. Biankin, R. Casolino

**Affiliations:** 1 General and Pancreatic Surgery Unit, Pancreas Institute, University of Verona, Verona; 2 Department of Environmental and Preventive Science, University of Ferrara, Ferrara; 3 Unit of Biostatistics, Epidemiology and Public Health, Department of Cardiac, Thoracic, Vascular Sciences, and Public Health, University of Padova, Padova, Italy; 4 Oncology Institute, Sheba Medical Center at Tel-Hashomer, Tel Aviv University, Tel Aviv, Israel; 5 Department of Surgery, New York University, New York; 6 Perlmutter Cancer Center, New York University, New York; 7 Department of Surgery and Surgical Oncology, Weill Cornell University, New York; 8 Englander Institute of Precision Medicine, Weill Cornell University, New York, USA; 9 Wolfson Wohl Cancer Research Centre, School of Cancer Sciences, University of Glasgow, Glasgow; 10 West of Scotland Pancreatic Unit, Glasgow Royal Infirmary, Glasgow, UK; 11 Faculty of Medicine, South Western Sydney Clinical School, University of NSW, Liverpool, Australia

**Keywords:** pancreatic cancer, BRCA, germline testing, disparities

## Abstract

**Background:**

Germline *BRCA1* and *BRCA2* mutations (gBRCAm) can inform pancreatic cancer (PC) risk and treatment but most of the available information is derived from white patients. The ethnic and geographic variability of gBRCAm prevalence and of germline BRCA (gBRCA) testing uptake in PC globally is largely unknown.

**Materials and methods:**

We carried out a systematic review and prevalence meta-analysis of gBRCA testing and gBRCAm prevalence in PC patients stratified by ethnicity**.** The main outcome was the distribution of gBRCA testing uptake across diverse populations worldwide. Secondary outcomes included: geographic distribution of gBRCA testing uptake, temporal analysis of gBRCA testing uptake in ethnic groups, and pooled proportion of gBRCAm stratified by ethnicity. The study is listed under PROSPERO registration number #CRD42022311769.

**Results:**

A total of 51 studies with 16 621 patients were included. Twelve of the studies (23.5%) enrolled white patients only, 10 Asians only (19.6%), and 29 (56.9%) included mixed populations. The pooled prevalence of white, Asian, African American, and Hispanic patients tested per study was 88.7%, 34.8%, 3.6%, and 5.2%, respectively. The majority of included studies were from high-income countries (HICs) (64; 91.2%). Temporal analysis showed a significant increase only in white and Asians patients tested from 2000 to present (*P* < 0.001). The pooled prevalence of gBRCAm was: 3.3% in white, 1.7% in Asian, and negligible (<0.3%) in African American and Hispanic patients.

**Conclusions:**

Data on gBRCA testing and gBRCAm in PC derive mostly from white patients and from HICs. This limits the interpretation of gBRCAm for treating PC across diverse populations and implies substantial global and racial disparities in access to BRCA testing in PC.

## Highlights

• gBRCA testing is relevant for precision PC treatment and more broad cancer screening strategies.
• Little is known about the ethnicity of PC patients tested for germline BRCA1/2 mutations.
• We show that most PC patients tested for germline BRCA1/2 mutations in research context are white (74.7%) and from high-income countries (91.2%).
• These findings implie major global and ethnic disparities in access to gBRCA testing for patients with PC.
• Global efforts are urgently needed to promote accessibility of non-white patients to gBRCA testing and, in general, to clinical trials.

## Introduction

Germline aberrations in *BRCA1* and *BRCA2* genes are prevalent and clinically relevant for treatment and potential screening for pancreatic cancer (PC).^1^, ^2^, ^3^ They are associated with increased progression-free survival in metastatic PC patients treated with poly-ADP-ribose polymerase inhibitor olaparib in the maintenance setting^4^ and confer better response to platinum-based chemotherapy.^5^, ^6^, ^7^, ^8^ Germline *BRCA1* and/or *BRCA2* mutations (gBRCAm) can also inform cancer risk and drive preventative strategies in healthy relatives of patients with PC who are at high risk of BRCA-associated cancers (i.e. breast cancer, ovarian cancer, prostate cancer, and PC).^9^, ^10^, ^11^, ^12^ Given the above implications, and the lack of significant family history of cancer in up to 60% of mutation carriers,^13^, ^14^, ^15^ the American Society of Clinical Oncology and the National Comprehensive Cancer Network now recommend *BRCA* germline (gBRCA) testing for all individuals with PC irrespective of personal or family history, age, or ethnicity, to maximize the opportunity for targeted treatments for patients and screening for families.^5^^,^^16^

Although such progress provides promising opportunities for a subset of patients with PC, most data that define our current understanding of the role and clinical relevance of gBRCAm in PC have been generated within the context of mainly European ancestry (commonly termed as ‘white’ or ‘Caucasians’).^15^^,^^17^ Lack of data from non-white populations, likely due to disparities in access to genetic testing in routine and research settings, limits our understanding of the clinical significance of gBRCAm in broad populations with PC. This limitation is consistent with the general under-representation of racial minorities in precision oncology studies broadly as well as genetic and genomic research.^18^, ^19^, ^20^, ^21^, ^22^ This is a major public health concern as it precludes access to innovative and potentially active oncological treatments, limits scientific progress by underappreciating genetic diversity, and translates into significant health disparities of already marginalized racial minorities.^21^, ^22^, ^23^, ^24^, ^25^, ^26^, ^27^, ^28^, ^29^, ^30^, ^31^, ^32^, ^33^, ^34^, ^35^, ^36^, ^37^

Studies of ethnic and geographic variability of gBRCA testing uptake and gBRCAm prevalence in PC are currently lacking. To the best of our knowledge, this aspect has only been described in the phase III POLO trial of maintenance olaparib in patients with advanced, platinum-sensitive PC carrying a gBRCAm.^38^ However, this study included a small number of non-white patients, making conclusions about the prevalence of gBRCAm in diverse populations challenging.

Here we carried out a systematic review and meta-analysis of the ethnic and geographic variability of gBRCA testing uptake and gBRCAm prevalence in patients with PC. Data concerning gBRCAm in PC are mostly from white patients and high-income countries (HICs), limiting interpretation of gBRCAm testing and associated therapy across diverse populations and highlighting disparities in access to testing and clinical trials.

## Materials and methods

### Search strategy, selection, and inclusion criteria

**Figure 1:**
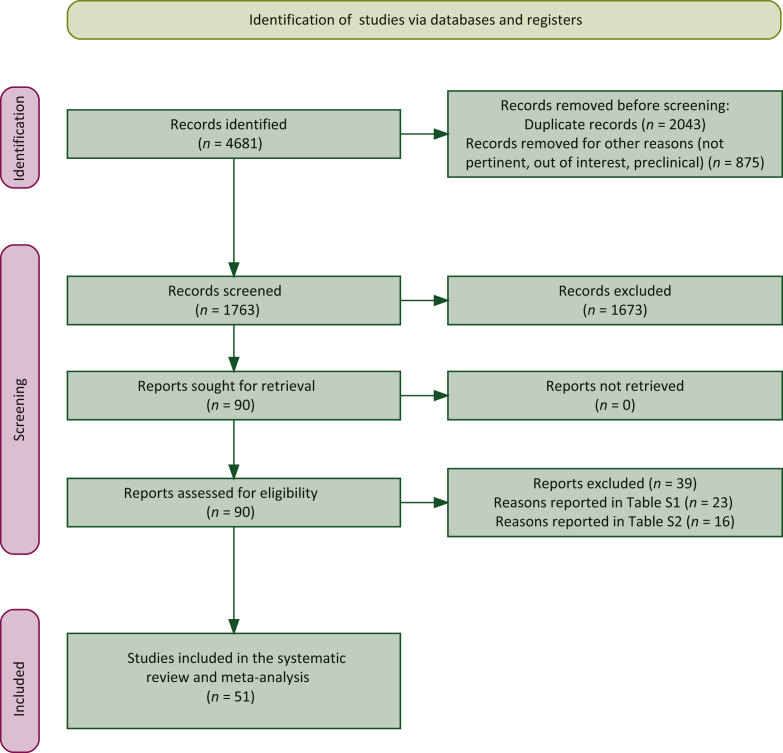
Preferred Reporting Items for Systematic reviews and Meta-Analyses (PRISMA) flowchart.

The study protocol and data extraction for the systematic review and meta-analysis were designed according to the updated version of the Preferred Reporting Items for Systematic reviews and Meta-Analyses (PRISMA) guidelines.^39^ The research protocol was registered at the International Prospective Register of Systematic Reviews (PROSPERO, https://www.crd.york.ac.uk/prospero/; registration number: #CRD42022311769). This systematic review was arranged as follows: (i) identification: to search studies on germline *BRCA1* and *BRCA2* testing in patients with PC; (ii) screening: to search all studies reporting the ethnicity of patients included; (iii) eligibility: to include all data reporting the prevalence of pathogenic and/or likely pathogenic gBRCAm stratified as per patients’ ethnicity; (iv) inclusion: to analyze the extracted information. The PRISMA flowchart is reported in Figure 1, while the PRISMA checklist can be found in Supplementary Material S1, available at https://doi.org/10.1016/j.esmoop.2023.100881.

PubMed, Scopus, and the Cochrane Library databases were queried for English language articles and reporting gBRCAm in patients with PC, published from January 2000 to 19 February 2022. Publicly available cancer genomic datasets (https://www.cbioportal.org/, https://portal.gdc.cancer.gov/, and https://dcc.icgc.org/) were also queried. A detailed search strategy is reported in Supplementary Material S1, available at https://doi.org/10.1016/j.esmoop.2023.100881.

Two authors independently screened the titles and abstracts of all identified articles (SP, RC). Articles were included if the study cohort was composed of at least 20 patients, regardless of study type and design. The authors worked independently, and each selected manuscript was double-checked. After the initial set of articles were identified, four authors resolved discrepancies through consensus (SP, RC, AVB, RS). Study selection and data extraction are presented in Supplementary Material S1, available at https://doi.org/10.1016/j.esmoop.2023.100881.

All authors had access to the study data and reviewed and approved the final manuscript.

### Outcomes of interest and data extraction

The main outcome was the pooled proportion of gBRCA testing uptake across all patients, with a focus on diverse ethnicities. Secondary outcomes were the pooled proportion of gBRCAm stratified as per ethnicity, the temporal analysis of gBRCA testing uptake in diverse populations, and the geographic distribution of gBRCA testing utilized worldwide.

For each study, the country of origin, study period, sample size, and patients’ ethnicity were collected. Only pathogenic or likely pathogenic mutations were extracted. Studies reporting gBRCA testing and results stratified as per patient’s ethnicity were considered for the prevalence analysis. Given the granularity of the data reported in literature, only the following ethnicities were considered: Caucasian/white, African American, Asian, Hispanic/Latino, mixed, minorities (e.g. Hawaiian/Pacific Islander). For the same reason, information on gBRCAm was collected overall, without distinction between familial and sporadic studies.

When ethnicity of the patient was not reported, raw data were requested from the corresponding authors. If no response was obtained, the manuscript was not entered into the systematic review. Studies with only partial information on ethnicity were not included in the systematic review. Studies focusing on Ashkenazi Jewish ancestry patients only were excluded as the prevalence of gBRCAm in this subgroup was previously described in our recent systematic review and meta-analysis.^15^

Countries are classified according to the World Bank as lower-middle-income country (LMIC), upper-middle-income country (UMIC), and HIC.^40^

### Research ethics

The concepts of race, ethnicity, or ancestry reported in the manuscript are not intended to discriminate patients/individuals, and they are merely considered for biomedical research purposes. The possible clinical and social consequences of this research were also evaluated.

The authors acknowledge race as a social construct rather than a biological category but given the lack of scientific validity of race for human categorization and the social implications associated to this term, the word ethnicity has been adopted instead.

### Statistical analysis

Descriptive statistics were used to quantify the frequencies of gBRCAm by patient ethnicity and frequencies of patient ethnicity tested, regardless of the testing result. A random-effects meta-analysis (DerSimonian and Laird model) was carried out on the prevalence data to calculate the pooled event rate using the Freeman–Tukey transformation.^41^^,^^42^ The study-specific and the pooled prevalence with the 95% confidence intervals (CIs) were graphically represented in a forest plot. The Cochran’s Q test for heterogeneity was carried out, reporting the I^2^ statistic, which indicates the percentage of variation across studies due to heterogeneity rather than chance.^43^ Heterogeneity values of >75% were classified considerable.^44^ A temporal analysis was made, dividing the study period into three groups: 2000-2009, 2009-2019, and after 2019 to present. The random-effects meta-regression models and subgroup analyses were estimated accounting for the study period before 2009, 2009-2019, and after 2019 as a possible effect modifier. Statistical analysis was carried out using R (R Foundation for Statistical Computing, Vienna, Austria, v. 4.02)^45^ with the metafor 2.4-0^46^ and FactoMineR packages.^47^

### Publication bias and study bias

The publication bias has been graphically inspected using a funnel plot representation where the inverse of standard error against log odds is shown. This representation has been considered because the literature demonstrated that the conventional funnel plots representing log odds versus the inverse of standard error could be asymmetric despite no publication bias for the event rate outcome.^48^

The risk of bias was assessed by using Translating-ROB (i.e. TRANSLATIonal caNcer Genomic Risk Of Bias), specifically developed for this kind of studies.^15^ A 25-point quality rating scale was applied to each study included in the systematic review (Supplementary Material S2, available at https://doi.org/10.1016/j.esmoop.2023.100881).

## Results

A total of 4681 articles were retrieved from electronic databases. Before abstract screening, 2918 papers were removed (duplicates, not pertinent, out of interest, preclinical studies). After screening abstracts and titles, 1673 studies were judged not relevant, with a total of 90 studies assessed for eligibility. After screening full texts, 23 studies were further excluded and 67 were evaluated for eligibility. Data on participant ethnicity were not available for 26 of the 67 articles. The corresponding authors of 10 of these 26 studies (38.5%) were contacted and they made available ethnic data, making their studies eligible for inclusion, while the remaining 15 studies were excluded due to lack of information. A total of 51 articles with 16 621 patients [median 133 patients/study, interquartile range (IQR) 247] were included in the final review and meta-analysis. The flowchart of the study selection process is reported in Figure 1 (PRISMA^39^ flowchart) while details on studies excluded are reported in Supplementary Material S1 and Tables S1 and S2, available at https://doi.org/10.1016/j.esmoop.2023.100881. Table 1 shows the general characteristics of included studies. With regard to the study design, 24 (47%) were retrospective, 14 (27.4%) were prospective, 11 (21.5%) were registry-based, and 2 (4.1%) were case-control studies.

**Table 1:** General features of the studies included in the systematic review and meta-analysis

Study	Year	Country	*N*	Study period	Study design	Stage	Race/ethnicity of patients enrolled	Main outcome meta-analysis^a^	Secondary outcome meta-analysis^b^
Yin et al.^70^	2022	China	1009	2006-2017	Case series	NR	Chinese (Han), 100%	Yes	Yes
Chittenden et al.^71^	2021	USA	266	2017-2019	P	Mixed	White, 92.8% Hispanic, 3.4% African American, 1.1% Other, 2.6%	Yes	No
Varghese et al.^72^	2021	USA	450	2008-2018	R	Mixed	White, 78.4% African American, 9.1% Asian, 5.8% Native/unknown/other, 6.7%	Yes	No
Shui et al.^73^	2021	China	195	2016-2019	R	Mixed	Asian, 100%	Yes	Yes
Walker et al.^74^	2021	USA	158	2018-2019	R	NR	White, 70% Asian, 16% Hispanic, 6% African American, 4% Multiethnic/other, 2% Unknown/declined, 2%	Yes	Yes
Uson et al.^75^	2021	USA	250	2018-2020	P	Mixed	White, 83.6% Hispanic, 6.8% African American, 5.2% Asian, 1.6% American Indian, 1.6% Native Hawaiian, 0.8% Other, 0.4%	Yes	No
Hata et al.^76^	2021	Japan	39	2017-2020	P	Mixed	Asian, 100%	Yes	Yes
Wieme et al.^77^	2021	Czech Republic/Belgium	298	2015-2018	Mixed	NR	White, 100%^c^	Yes	Yes
Fountzilas et al.^78^	2021	Greece/Cyprus	549	NR	R	Mixed	White, 100%	Yes	Yes
Zimmermann et al.^79^	2021	USA	535	2009-2017	P	Mixed	White, 89% African American, 7% Hispanic, 2% Asian, 2% Other, 1% Native American, <1%	Yes	No
Takai et al.^80^	2020	Japan	81	2002-2015	R	NR	Japanese, 100%	Yes	Yes
Earl et al.^81^	2020	Spain	43	NR	R-B	NR	White, 100%	Yes	Yes
Krepline et al.^82^	2020	USA	127	2009-2018	R	Localized	White, 92%, African American, 4% Hispanic, 2% Other, 2%	Yes	No
Mizukami et al.^83^	2020	Japan	1005	2003-2018	R	Mixed	Asian, 100%	Yes	Yes
Park et al.^84^	2020	USA	262	2013-2019	P	Adv	White, 82% African American, 6.1% Asian, 5.7% Unknown, 5.7%	Yes	No
Cremin et al.^85^	2020	Canada	177	2016-2019	P	Mixed	White, 70.1% Asian, 21.5% Ashkenazi Jewish, 1.7% Other, 4% Missing, 2.8%	Yes	No
Golan et al.^17^	2020	International	2154	2015-2019	P	Adv	White, 85.3% Asian, 10.5% African American, 1.3% Other, 3%	Yes	Yes
Goldstein et al.^86^	2020	USA	133	2010-2016	R	Adv	White, 83.4% African American, 11.3% Hispanic, 5.3% Asian, 1.5% Other, 1.5%	Yes	Yes
McIntyre et al.^87^	2020	USA	283	2004-2017	P	Mixed	White, 88% Hispanic, 5% Asian, 4% African American, 2% Unknown, 1%	Yes	No
Bertelsen et al.^88^	2019	Denmark	43	2013-2018	P	Adv	White, 100%	Yes	Yes
Yurgelun et al.^89^	2019	USA	289	2002-2013	P	Res	White, 76% Asian, 10% African American, 1% Unknown, 13%	Yes	No
Palacio et al.^90^	2019	USA	40	2012-2018	R	Adv	Hispanic, 70% White, 25% Asian, 1% African American, 0% Other, 1%	Yes	No
Takeuchi et al.^91^	2018	Japan	42	2007-2014	R	Res	Asian, 100%	Yes	Yes
Bannon et al.^92^	2018	USA	277	2005-2016	R	Mixed	White, 82.7% African American, 7.2% Hispanic, 6.8% Asian, 3% Unknown, <1%	Yes	No
Chaffee et al.^93^	2018	USA	302	2000-2013	R	Mixed	White, 97% African American, 1,3% American Indian, 0.3% Hawaiian, 0.3% Multiracial, 0.3% Missing, 0.7%	Yes	Yes
Ohmoto et al.^94^	2018	Japan	20	2007-2013	R	Mixed	Asian, 100%	Yes	Yes
Smith et al.^95^	2018	Canada	386	2014-2016	R-B	Mixed	White, 100% —Ashkenazi Jewish, 9.4%	Yes	Yes
Slavin et al.^96^	2018	USA–Latin America	53	1996-2016	R-B	NR	White, 77% Hispanic, 11% Asian, 9% American Indian, 2%	Yes	No
Sehdev et al.^97^	2018	USA	36	NR	R	Res	White, 97.2% African American, 2.8%	Yes	No
Lowery et al.^14^	2018	USA	615	2014-2017	P	Mixed	White, 89.4% —Non-Ashkenazi Jewish, 79.8% —Ashkenazi Jewish, 20.2% Black/Hispanic, 6% Asian, 4.6%	Yes	No
Kondo et al.^98^	2018	Japan	28	2015-2017	P	Mixed	Asian, 100%	Yes	Yes
Shahda et al.^99^	2018	USA	57	NR	R	Adv	White, 83% African American, 17%		
Hu et al.^100^	2018	USA	3030	2000-2016	C-C	Mixed	White, 95.6% African American, 1.6% Hispanic, 1.4% Asian, 0.4% Other, 0.6% Missing, 0.4%	Yes	Yes
Brand et al.^101^	2018	USA	298	2015-2016	R	Mixed	White, 85.9% Ashkenazi Jewish, 8.7% African American, 3.7% Hispanic, 0.7% Asian, 0.3% Multiple/other/unknown, 0.7%	Yes	Yes
Aung et al.^102^	2018	Canada	63	2015-2017	P	LA	White, 70% Asian, 29% African American, 1%	Yes	No
Macklin et al.^103^	2018	USA	59	2012-2018	R	NR	White, 86.4%^c^ African American, 10.2% Hispanic, 1.7% Unknown, 1.7%	Yes	Yes
Alimirzaie et al.^104^	2018	Iran	24	2011-2014	P	Mixed	White, 100%	Yes	Yes
Aguirre et al.^105^	2018	USA	71	2015-2017	P	Adv	White, 80% African American, 10% Other, 10%	Yes	No
Shindo et al.^106^	2017	USA	854	2000-2015	R	Res	White, 89% African American, 6% Other, 5%	Yes	Yes
Connor et al.^107^	2017	International	154	2008-2015	R	Mixed	Unknown, 48.7%^c^ White, 42.2% Ashkenazi Jewish, 3.2% Asian, 2.6% African American, 1.3% Mixed, 1.3% Hispanic, 0.6%	Yes	Yes
Takai et al.^108^	2016	Japan	54	2002-2013	R	Adv	Asian, 100%	Yes	Yes
Roberts et al.^109^	2016	USA	638	NR	R-B	NR	White, 96% African American, 2.8% Asian, 1.2%	Yes	No
Zhen et al.^110^	2015	USA	717	NR	R-B	NR	White, 87.3% Multiracial, 7.1% African American, 2.8% Asian/Asian American, 1.2% American Indian/Native, 0.2% Other, 1.4%	Yes	No
Lucas et al.^111^	2014	USA	32	2005-2011	R-B	NR	White, 100%	Yes	Yes
Ghiorzo et al.^112^	2012	Italy	29	1999-2011	P	NR	White, 100%	Yes	Yes
Axilbund et al.^113^	2009	USA	66	NR	R-B	NR	White, 93.9% African American, 3% Hispanic, 1.5% Other, 1.5%	Yes	Yes
Lawniczak et al.^114^	2008	Poland	62	2002-2007	P	Mixed	White, 100%	Yes	Yes
Cho et al.^115^	2008	South Korea	60	1998-2002	P	NR	Asian, 100%	Yes	Yes
Hahn et al.^116^	2003	Germany–UK	64^d^	1999-2002	R-B	NR	White, 100%	Yes	Yes
Murphy et al.^117^	2002	USA	29	1994-2001	R-B	NR	White, 100%	Yes	Yes
Real et al.^118^	2002	Spain	72	1992-1995	P	NR	White, 100%	Yes	Yes

Adv, advanced; C-C, case-control; gBRCAm, germline BRCA1 and BRCA2 mutations; LA, locally advanced; Mix, mixed; NR, not reported; P, prospective; R, retrospective; R-B, registry-based; Res, resected.

a Prevalence meta-analysis of patients’ ethnicities.

b Pooled proportion of gBRCAm stratified as per patients’ ethnicity.

c Data obtained upon authors’ contact. The request was addressed to those studies not reporting race/ethnicity at all.

d Families.

**Table 2:** Prevalence meta-analysis

	Studies (*n*)	Pooled prevalence (95% CI)	Het. (I^2^)	Studies (*n*)	Pooled prevalence (95% CI)	Het. (I^2^)	Studies (*n*)	Pooled prevalence (95% CI)	Het. (I^2^)	Studies (*n*)	Pooled prevalence (95% CI)	Het. (I^2^)
	White patients			African American patients			Asian patients			Hispanic patients		
Tested	41	88.7% (83.4% to 93.2%)	100%	26	3.6% (2.5% to 4.9%)	91%	30	34.8% (16.1% to 56.4%)	100%	16	5.2% (1.7% to 10.2%)	100%
Any BRCA mutation	21	3.3% (1.7% to 5.3%)	84%	8	0.2% (0% to 0.7%)	64%	15	1.7% (0.3% to 3.9%)	93%	6	0% (0% to 0%)	0%
BRCA1 mutations	20	0.3% (0% to 0.9%)	72%	5	0% (0% to 0%)	2%	13	0% (0% to 0.1%)	23%	—a	—	—
BRCA2 mutations	19	2.2% (1.1% to 3.6%)	76%	5	0% (0% to 0.1%)	14%	13	2% (0.4% to 4.5%)	91%	—a	—	—

CI, confidence interval; Het., heterogenicity.

a Not carried out due to the low number of studies included (≤3).

### Main outcome measure

**Figure 2:**
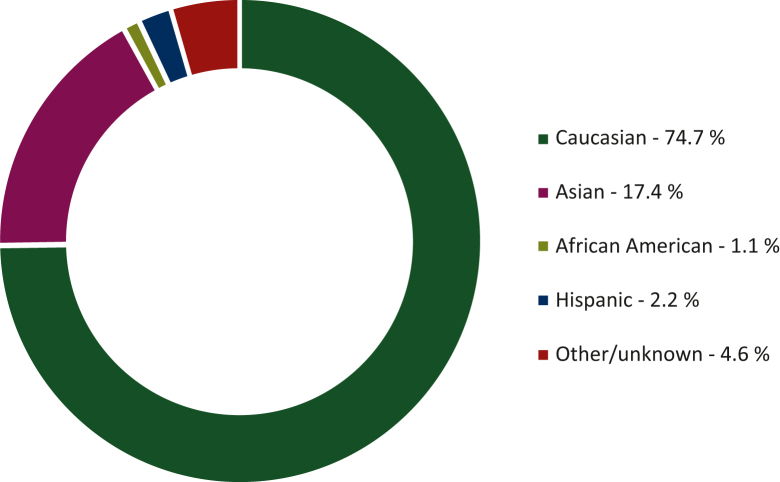
Overview of ethnicities in included studies. White patients accounted for 74.7% of all patients enrolled in the 51 studies included in the systematic review and meta-analysis, followed by Asians (17.4%). African Americans and Hispanics were definitely under-tested (1.1% and 2.2%, respectively); the remaining 4.6% gathers uncommon ethnicities (e.g. Hawaiian, Native Americans), Ashkenazi Jewish descendants, mixed ethnicities, and cases of unknown ancestry.

Of the 51 studies included in the systematic review and meta-analysis, 12 (23.5%) enrolled white patients only, 10 Asians only (19.6%), while the remaining 29 (56.9%) included mixed populations. White patients accounted for the 74.7% of all patients included, followed by Asians (17.4%). African Americans and Hispanics were under-represented (1.1% and 2.2%, respectively); the remaining 4.6% included uncommon ethnicities (e.g. Hawaiian, Native Americans), Ashkenazi Jewish descendants, mixed ethnicities, and cases of unknown ancestry (Figure 2; Table 1).

A total of 29 studies including mixed populations and reporting gBRCA testing uptake stratified by ethnicity were considered for the prevalence analysis. The pooled proportion of ethnicities per study for white, Asian, African American, and Hispanic patients was 88.7% (95% CI 83.4% to 93.2%; I^2^ 100%), 34.8% (95% CI 16.1% to 56.4%; I^2^ 100%), 3.6% (95% CI 2.5% to 4.9%; I^2^ 91%), and 5.2% (95% CI 1.7% to 10.2%; I^2^ 90%), respectively (Supplementary Material S3, available at https://doi.org/10.1016/j.esmoop.2023.100881).

### Secondary outcomes

Thirty-two out of 51 studies (62.7%), including 11 395 patients (median 69 patients/study, IQR 257), reported results of gBRCA testing stratified by ethnicity. The pooled proportion of any gBRCAm was: 3.3% (95% CI 1.7% to 5.3%) for white, 1.7% (95% CI 0.3% to 3.9%) for Asian, 0.2% (95% CI 0% to 0.7%) for African American, and 0% (95% CI 0% to 0%) for Hispanic patients (Supplementary Material S3, available at https://doi.org/10.1016/j.esmoop.2023.100881, and Table 2). Less represented ethnicities were excluded from the meta-analysis due to granularity of data (Table 2).

**Figure 3:**
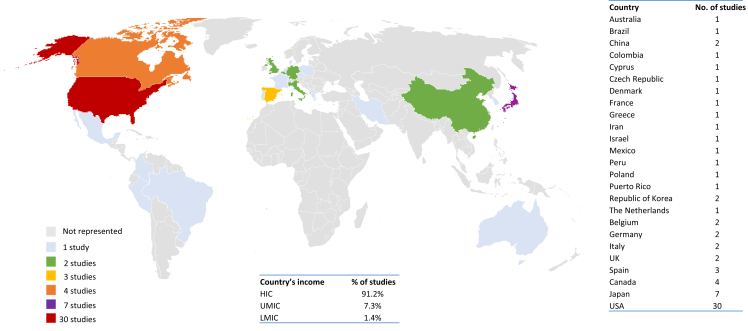
Geographic distribution of studies on germline *BRCA* in pancreatic cancer. Overview of the geographic distribution of studies included in the systematic review and meta-analysis. The table shows the number of studies per country, including both those conducted in individual countries and international studies. Countries are classified according to the World Bank as lower-middle-income country (LMIC), upper-middle-income country (UMIC), and high-income country (HIC) (https://datahelpdesk.worldbank.org/knowledgebase/articles/906519).

The continental distribution of the studies was: 8 from Europe (15.7%), 30 from North America (58.8%; 27 from USA, 3 from Canada), 1 combined from USA and Latin America, and 10 from Asia (21.5%). Two studies (3.9%) were generated by intercontinental collaborations (Figure 3). Considering both studies conducted in individual countries and international collaborations, the vast majority of study sites were in HICs (65; 91.2%), while UMICs and LMICs were poorly represented (5; 7.3% and 1; 1.4%, respectively) (Figure 3).

The temporal analysis showed a statistically significant increase in the percentage of white and Asian patients tested over time only (test for subgroup differences, both *P* < 0.001*,* Supplementary Material S3, available at https://doi.org/10.1016/j.esmoop.2023.100881) while for the other ethnicities this was not significant.

The risk of bias assessment through Translating-ROB, based on a 25-point scale, showed a median value of the rating score of 20, IQR 6 (Supplementary Material S2, available at https://doi.org/10.1016/j.esmoop.2023.100881).

## Discussion

This is the first systematic review and meta-analysis describing the global landscape of gBRCAm in PC with particular focus on ethnic and geographic variability in utility of genetic testing and overview of pathogenic mutation prevalence. Our findings show that, similar to all genetic knowledgebases, data on gBRCAm in PC derive mostly from white patients (74.7%) and from HICs (91.2%), while those from minority populations and limited-resource countries are extremely low. This is in line with recent reports on the ethnicity of PC patients participating in clinical trials, showing that 84.7% of patients enrolled are of white origin,^21^ and with the findings from the POLO trial.^17^ Poor representation of minorities in clinical trials and genomic research also applies to other common cancers^18^^,^^49^^,^^50^ reflecting substantial inequalities in accessing medical research.^33^^,^^34^

The reasons for this are multifactorial, and explanation is beyond this study’s aims; however, some considerations can be made according to the included geographic regions of our included studies. Thirty studies originated from North America (the USA and Canada, and one collaboration USA/Central-Latin America), including 10 528 PC patients overall. The prevalence of non-Hispanic white patients tested was 74.7%, leaving the remaining 25.3% for all other ethnicities. This is despite the higher PC rate in non-Hispanic black patients, compared to non-Hispanic white patients (16.3 versus 14.1 per 100 000, respectively).^51^ The population in the United States is multiracial, where many genetic ancestries coexist.^52^ According to the United States Census Bureau 2020 results, African Americans, Asians, and Hispanics account for approximately 12.4%, 6%, and 16.3% of the population, respectively (including other ethnicities, they constitute 39% of the population).^53^ This ethno-racial heterogeneity is not reflected in the present study findings, where the rate of gBRCA testing uptake in non-white patients was extremely low. In fact, the disproportionate bias of higher rates in the non-Hispanic black population exposes a severe under-engagement of the actual patient pool/catchments. The payor coverage of genetic testing in the United States, which is based on health insurance plans, is certainly one of the main reasons for ethnic disparities in access to BRCA testing here observed.

Eight studies originated from Europe, where the ethno-geographic makeup is more complex, granular, and country-specific.^54^ It is estimated that 2% of the European population has African ancestry,^55^ and according to the World Health Organization, almost 10% of the European population are international migrants.^56^ Based on the results of the current study, we estimated that thousands of PC patients of non-white origin are realistically excluded from medical studies. Of note, none of the studies included in the present analysis originated from any Eastern European nation.^57^

Asian countries and Asian patients were also under-represented. While Asians represented the second most common ethnicity (17.4% of the patients included), this number is extremely low when considering the entire population of countries such as China or the Indian subcontinent (that are not represented at all). Sociocultural, structural, and economic barriers may explain this extremely low access to gBRCA testing in Asian countries, and no clear conclusions can be drawn. Notably, 7 out of 10 studies including Asian patients were from Japan, an HIC, further suggesting economic factors as a key driver of disparities in genomic research between same continent countries with different income.

Another important aspect that emerged from the present study is the identification of poor reporting of ethnicity amongst the studies included. Twenty-six out of 67 potentially eligible studies, corresponding to 38.8%, did not provide any information about participant ethnicity. Excluding those conducted in mono-ethnic populations only (*n* = 22; 12 in white, 10 in Asians), this rate rises to 57.7%. When it comes to reporting gBRCAm prevalence, only 8 of 29 studies enrolling mixed populations reported ethnicity information (27.6%). This indicates significant bias in reporting methods and results and lesser importance attributed to ethnicity in medical research. The general poor reporting of studies has also been pointed out by the risk of bias assessment through Translating-ROB. The median value of the rating score based on a 25-point scale was 20, IQR 6 (no study reached the maximum of 25). The reasons for significant inaccuracy in reporting studies are unknown and may not be limited to studies focused on PC.

Last, we identified substantial discrepancies in the pooled prevalence of gBRCAm in different ethnic groups (3.3% in white, 1.7% in Asians, 0.2% in African Americans, and 0% in Hispanics). However, this needs to be interpreted with caution. Indeed, given the overall low prevalence of gBRCAm in unselected PC patients, and the scarce inclusion of non-white patients in the current study, no clear conclusions can be drawn on any real difference in the prevalence of gBRCAm across populations. Enhancing diversity and equity in genomic research will be essential to define the real prevalence of gBRCAm in non-white populations and to assess any significant differences in mutation rate across ethnicities, all as an effect to more effectively mitigate cancer risk and to optimize personalized treatment of patients.

The current lack of diversity identified in BRCA research is of concern as it does not assess human variability,^31^^,^^32^ thus limiting the generalizability of research findings that may not account for differences in biological and sociocultural factors across populations impacting PC susceptibility and treatment outcomes.^58^, ^59^, ^60^, ^61^, ^62^, ^63^, ^64^, ^65^, ^66^ For *BRCA1* and *BRCA2* genes, founder mutations have been identified across multiple ethnicities,^67^^,^^68^ including African Americans and Hispanics, only seldom profiled in PC due to global and racial disparities in BRCA testing uptake.^26^^,^^27^ This will likely compound the already existing inequities in cancer care described for patients with PC,^23^, ^24^, ^25^ which translates to poorer survival amongst non-white populations, especially African Americans.^26^^,^^28^, ^29^, ^30^ Multi-level interventions are encouraged to enhance inclusion of multiple ethnicities in PC genomic studies to better understand cancer susceptibility across diverse populations and improve tailored early detection strategies, to allow minority groups access to innovative targeted treatments and, more in general, to mitigate health disparities in cancer screening and treatment.

This study has some limitations, including: (i) general heterogeneity among studies in terms of sample size, study period, patients enrolled (familial or sporadic cases), and study design; (ii) inconsistency of definitions used to categorize human populations in different studies (race, ethnicity, or ancestry, often interchangeably used^33^), making results only partially comparable; (iii) general low numbers of non-white PC patients, limiting the validity of the pooled estimates of gBRCAm in these subgroups; (iv) lack of investigation of factors that may influence access to genetic testing in included studies; (v) considerable heterogeneity in some meta-analysis results; and (vi) inherent publication bias and study bias (e.g. overlapping study populations coming from the same institution). Altogether, these factors hamper analysis and interpretation.

In conclusion, although gBRCA testing is relevant for precision PC treatment and more broad cancer screening strategies, this study suggests that information on gBRCAm in PC derives mostly from white patients and from studies conducted in HICs. This implies racial and global disparities in access to BRCA testing for patients with PC and translates into missed opportunities: (i) to study ethnic and racial minorities in terms of impact of social determinants of cancer risk and survival outcomes; (ii) to expand effective precision medicine strategies globally, including PC prevention and treatment; and (iii) to enhance the understanding of cancer biology in minority populations and pharmacoethnicity.^69^ Real-world data will be important to see if this figure reflects access to genetic testing as standard of care, outside the research setting. Our findings suggest that there is an urgent need for a concerted effort to improve global access to BRCA testing in PC and, in general, address genomic diversity as well as geographic and racial disparities in research and health care delivery.

## Supplementary data

Supplementaty Material 1

Supplementaty Material 2

Supplementaty Material 3

Supplementaty Material 4
