# Supplementary material for: A systematic review and meta-analysis of germline BRCA mutations in pancreatic cancer patients identifies global and racial disparities in access to genetic testing
Source: ESMO Open. 2023 Feb 21;8(2):100881. doi: 10.1016/j.esmoop.2023.100881 (PMC10163165; doi:10.1016/j.esmoop.2023.100881)
Supplement: Supplementaty Material 1 [file mmc1.docx]

***Supplementary material 1***

***PRISMA® checklist^1^***

| **Section and Topic** | **Item #** | **Checklist item** | **Location where item is reported** |
| --- | --- | --- | --- |
| **TITLE** | | |  |
| Title | 1 | Identify the report as a systematic review. | Title page |
| **ABSTRACT** | | |  |
| Abstract | 2 | See the PRISMA 2020 for Abstracts checklist. | Abstract |
| **INTRODUCTION** | | |  |
| Rationale | 3 | Describe the rationale for the review in the context of existing knowledge. | Pg 5 |
| Objectives | 4 | Provide an explicit statement of the objective(s) or question(s) the review addresses. | Pg 5 |
| **METHODS** | | |  |
| Eligibility criteria | 5 | Specify the inclusion and exclusion criteria for the review and how studies were grouped for the syntheses. | Pg 6 |
| Information sources | 6 | Specify all databases, registers, websites, organisations, reference lists and other sources searched or consulted to identify studies. Specify the date when each source was last searched or consulted. | Pg 6-7 |
| Search strategy | 7 | Present the full search strategies for all databases, registers and websites, including any filters and limits used. | Pg, 6 and S1 |
| Selection process | 8 | Specify the methods used to decide whether a study met the inclusion criteria of the review, including how many reviewers screened each record and each report retrieved, whether they worked independently, and if applicable, details of automation tools used in the process. | Pg 6-7 |
| Data collection process | 9 | Specify the methods used to collect data from reports, including how many reviewers collected data from each report, whether they worked independently, any processes for obtaining or confirming data from study investigators, and if applicable, details of automation tools used in the process. | Pg 6-7 |
| Data items | 10a | List and define all outcomes for which data were sought. Specify whether all results that were compatible with each outcome domain in each study were sought (e.g. for all measures, time points, analyses), and if not, the methods used to decide which results to collect. | Pg 7 |
|  | 10b | List and define all other variables for which data were sought (e.g. participant and intervention characteristics, funding sources). Describe any assumptions made about any missing or unclear information. | Pg 7 |
| Study risk of bias assessment | 11 | Specify the methods used to assess risk of bias in the included studies, including details of the tool(s) used, how many reviewers assessed each study and whether they worked independently, and if applicable, details of automation tools used in the process. | Pg 8 |
| Effect measures | 12 | Specify for each outcome the effect measure(s) (e.g. risk ratio, mean difference) used in the synthesis or presentation of results. | Pg 7 |
| Synthesis methods | 13a | Describe the processes used to decide which studies were eligible for each synthesis (e.g. tabulating the study intervention characteristics and comparing against the planned groups for each synthesis (item #5)). | NA |
|  | 13b | Describe any methods required to prepare the data for presentation or synthesis, such as handling of missing summary statistics, or data conversions. | NA |
|  | 13c | Describe any methods used to tabulate or visually display results of individual studies and syntheses. | NA |
|  | 13d | Describe any methods used to synthesize results and provide a rationale for the choice(s). If meta-analysis was performed, describe the model(s), method(s) to identify the presence and extent of statistical heterogeneity, and software package(s) used. | Pg 7-8 |
|  | 13e | Describe any methods used to explore possible causes of heterogeneity among study results (e.g. subgroup analysis, meta-regression). | Pg 8 |
|  | 13f | Describe any sensitivity analyses conducted to assess robustness of the synthesized results. | NA |
| Reporting bias assessment | 14 | Describe any methods used to assess risk of bias due to missing results in a synthesis (arising from reporting biases). | Pg 8 |
| Certainty assessment | 15 | Describe any methods used to assess certainty (or confidence) in the body of evidence for an outcome. | NA |
| **RESULTS** | | |  |
| Study selection | 16a | Describe the results of the search and selection process, from the number of records identified in the search to the number of studies included in the review, ideally using a flow diagram. | Pg 8 |
|  | 16b | Cite studies that might appear to meet the inclusion criteria, but which were excluded, and explain why they were excluded. | S1 |
| Study characteristics | 17 | Cite each included study and present its characteristics. | Table 1 |
| Risk of bias in studies | 18 | Present assessments of risk of bias for each included study. | Pg 8, S2 |
| Results of individual studies | 19 | For all outcomes, present, for each study: (a) summary statistics for each group (where appropriate) and (b) an effect estimate and its precision (e.g. confidence/credible interval), ideally using structured tables or plots. | Pg 8-9 |
| Results of syntheses | 20a | For each synthesis, briefly summarise the characteristics and risk of bias among contributing studies. | S2 |
|  | 20b | Present results of all statistical syntheses conducted. If meta-analysis was done, present for each the summary estimate and its precision (e.g. confidence/credible interval) and measures of statistical heterogeneity. If comparing groups, describe the direction of the effect. | NA |
|  | 20c | Present results of all investigations of possible causes of heterogeneity among study results. | NA |
|  | 20d | Present results of all sensitivity analyses conducted to assess the robustness of the synthesized results. | NA |
| Reporting biases | 21 | Present assessments of risk of bias due to missing results (arising from reporting biases) for each synthesis assessed. | NA |
| Certainty of evidence | 22 | Present assessments of certainty (or confidence) in the body of evidence for each outcome assessed. | NA |
| **DISCUSSION** | | |  |
| Discussion | 23a | Provide a general interpretation of the results in the context of other evidence. | Pg 10-13 |
|  | 23b | Discuss any limitations of the evidence included in the review. | Pg 12 |
|  | 23c | Discuss any limitations of the review processes used. | Pg 12 |
|  | 23d | Discuss implications of the results for practice, policy, and future research. | Pg 11-13 |
| **OTHER INFORMATION** | | |  |
| Registration and protocol | 24a | Provide registration information for the review, including register name and registration number, or state that the review was not registered. | Pg 6 |
|  | 24b | Indicate where the review protocol can be accessed, or state that a protocol was not prepared. | NA |
|  | 24c | Describe and explain any amendments to information provided at registration or in the protocol. | NA |
| Support | 25 | Describe sources of financial or non-financial support for the review, and the role of the funders or sponsors in the review. | Pg 2-3 |
| Competing interests | 26 | Declare any competing interests of review authors. | Pg 2 |
| Availability of data, code and other materials | 27 | Report which of the following are publicly available and where they can be found: template data collection forms; data extracted from included studies; data used for all analyses; analytic code; any other materials used in the review. | Pg 3 |

***Search strategy***

RC and SP designed the study protocol and data extraction according to Preferred Reporting Items for Systematic reviews and Meta-Analyses (PRISMA) guidelines^1^. The research protocol was registered at the International Prospective Register of Systematic Reviews (PROSPERO, https://www.crd.york.ac.uk/prospero/; registration number: # CRD42022311769).

The following databases were searched from January 2000 to February 19, 2022:

- - PubMed/MEDLINE
  - Scopus
  - Cochrane Database of Systematic Reviews
  - Cochrane Central Register of Controlled Trials (source: Embase, CT.gov, PubMed, WHO *International Clinical Trials Registry Platform*)

The reference lists of all relevant articles were consulted to identify further pertinent studies. Hand searching was also done to identify studies that may not have been indexed in the databases.

**Publicly online-accessible cancer genomic datasets** reporting published, and unpublished data were also quired and considered for inclusion:

- The National Cancer Institute’s (NCI's) Genomic Data Commons (<https://portal.gdc.cancer.gov/>)
- International Cancer Genome Consortium (ICGC) data portal (<https://dcc.icgc.org/>)
- The cBioPortal for Cancer Genomics (<https://www.cbioportal.org/>)

*Search Strings*

*Pubmed*

((("pancreatic cancer"[All Fields] OR "pancreatic tumour"[All Fields]) OR "pancreatic adenocarcinoma"[All Fields]) OR "Pancreas cancer"[All Fields]) AND ((((((("BRCA"[All Fields] AND "DNA repair"[All Fields]) OR "DDR"[All Fields]) OR "homologous recombination"[All Fields]) OR "HRD"[All Fields]) OR "genomic"[All Fields]) OR "molecular profiling"[All Fields]) OR "molecular characterization"[All Fields])

*Scopus*

TITLE-ABS-KEY ( "pancreatic cancer" OR "pancreatic tumour" OR "pancreatic adenocarcinoma" OR "Pancreas cancer" ) AND ALL ( "BRCA" OR "DNA repair" OR "DDR" OR "genomic" OR "molecular profiling" OR "molecular characterization" )

*Cochrane*

("pancreatic cancer" OR "pancreatic tumour" OR "pancreatic adenocarcinoma" OR "Pancreas cancer" ) AND ( "BRCA" OR "DNA repair" OR "DDR" OR "genomic" OR "molecular profiling" OR "molecular characterization")

**Table 1S1. Studies excluded after full text reading (n= 23)**

| # | Study Title | First Author | Year | Reasons for exclusion |
| --- | --- | --- | --- | --- |
| 1 | Uptake and acceptability of a mainstreaming model of hereditary cancer multigene panel testing among patients with ovarian, pancreatic, and prostate cancer^2^ | Hamilton JG | 2021 | Out of interest |
| 2 | Yield and Utility of Germline Testing Following Tumor Sequencing in Patients With Cancer^3^ | Lincoln SE | 2020 | Out of interest |
| 3 | Overall survival in patients with pancreatic cancer receiving matched therapies following molecular profiling: a retrospective analysis of the Know Your Tumor registry trial^4^ | Pishvaian MJ | 2020 | Out of interest |
| 4 | Support of a molecular tumour board by an evidence- based decision management system for precision oncology^5^ | Lamping M | 2020 | Out of interest |
| 5 | Outcomes in Patients With Pancreatic Adenocarcinoma With Genetic Mutations in DNA Damage Response Pathways: Results From the Know Your Tumor Program^6^ | Pishvaian MJ | 2019 | Data on BRCA1/2 mutations not reported in detail |
| 6 | Molecular profiling of cancer patients enables personalized combination therapy: the I-PREDICT study^7^ | Sicklick JK | 2019 | Out of interest |
| 7 | Feasibility of personalized treatment concepts in gastrointestinal malignancies: Sub-group results of prospective clinical phase II trial EXACT^8^ | Unseld M | 2018 | Small sample size |
| 8 | Implementing a comprehensive translational oncology platform: from molecular testing to actionability^9^ | Mitri ZI | 2018 | Out of interest |
| 9 | Relevance of a molecular tumor board (MTB) for patients’ enrolment in clinical trials: experience of the Institut Curie^10^ | Basse C | 2018 | Out of interest |
| 10 | Integrated Genomic Characterization of Pancreatic Ductal Adenocarcinoma^11^ | Raphael BJ | 2017 | Out of interest |
| 11 | Clinical benefit of a precision medicine based approach for guiding treatment of refractory cancers^12^ | Radovich M | 2016 | Small sample size |
| 12 | Mutation analysis of the PALB2 gene in unselected pancreatic cancer patients in the Czech Republic^13^ | Borecka M | 2016 | Out of interest |
| 13 | Personalized Medicine in the Oncology Clinic: Implementation and Outcomes of the Johns Hopkins Molecular Tumor Board^14^ | Dalton WB | 2015 | Small sample size |
| 14 | Whole genomes redefine the mutational landscape of pancreatic cancer^15^ | Waddell N | 2015 | Out of interest |
| 15 | Molecularly targeted therapy based on tumour molecular profiling versus conventional therapy for advanced cancer (SHIVA): a multicentre, open-label, proof-of-concept, randomised, controlled phase 2 trial^16^ | Le Tourneau C | 2015 | Small sample size |
| 16 | Identification of germline genetic mutations in patients with pancreatic cancer^17^ | Salo-Mullen EE | 2015 | Ashkenazi Jewish only reported |
| 17 | Prevalence of germline mutations in cancer predisposition genes in patients with pancreatic cancer^18^ | Grant R | 2015 | Ashkenazi Jewish only reported |
| 18 | Personalized Medicine for Patients with Advanced Cancer in the Phase I Program at MD Anderson: Validation and Landmark Analyses^19^ | Tsimberidou AM | 2014 | Out of interest |
| 19 | High prevalence of BRCA1 and BRCA2 germline mutations with loss of heterozygosity in a series of resected pancreatic adenocarcinoma and other neoplastic lesions^20^ | Lucas AL | 2013 | Ashkenazi Jewish patients only |
| 20 | Routine testing for PALB2 mutations in familial pancreatic cancer families and breast cancer families with pancreatic cancer is not indicated^21^ | Harinck F | 2012 | Out of interest |
| 21 | Pilot Study Using Molecular Profiling of Patients' Tumors to Find Potential Targets and Select Treatments for Their Refractory Cancers^22^ | Von Hoff DD | 2010 | Small sample size |
| 22 | BRCA germline mutations in Jewish patients with pancreatic adenocarcinoma^23^ | Ferrone CR | 2009 | Ashkenazi Jewish patients only |
| 23 | The rate of the 6174delT founder Jewish mutation in BRCA2 in patients with non-colonic gastrointestinal tract tumours in Israel^24^ | Figer A | 2000 | Ashkenazi Jewish patients only |

**Table 2S1. Studies excluded due to data unavailability (n =16)**

| # | Study Title | First Author | Year | Reasons for exclusion |
| --- | --- | --- | --- | --- |
| 1 | Oncology clinic-based germline genetic testing for exocrine pancreatic cancer enables timely return of results and unveils low uptake of cascade testing^25^ | Wang Y | 2021 | Partially reported. Authors not contacted. |
| 2 | Pancreas cancer and BRCA: A critical subset of patients with improving therapeutic outcomes^26^ | Momtaz P | 2021 | Not reported. Authors contacted: not responding. |
| 3 | Germinal BRCA1-2 pathogenic variants (gBRCA1-2 pv) and pancreatic cancer: epidemiology of an Italian patient cohort ^27^ | Peretti U | 2021 | Not reported. Authors contacted: data not available. |
| 4 | A step towards personalizing next line therapy for resected pancreatic and related cancer patients: A single institution's experience^28^ | Lowder CY | 2020 | Not reported. Authors contacted: data not available. |
| 5 | Association of Germline Variants in Human DNA Damage Repair Genes and Response to Adjuvant Chemotherapy in Resected Pancreatic Ductal Adenocarcinoma^29^ | Hu H | 2020 | Partially reported. Authors not contacted. |
| 6 | Effect of Germline Mutations in Homologous Recombination Repair Genes on Overall Survival of Patients with Pancreatic Adenocarcinoma^30^ | Yadav S | 2020 | Partially reported. Authors not contacted. |
| 7 | Familial pancreatic adenocarcinoma: A retrospective analysis of germline genetic testing in a French multicentre cohort^31^ | Schwartz M | 2019 | Not reported. Authors contacted: data not available. |
| 8 | Real-Time Targeted Genome Profile Analysis of Pancreatic Ductal Adenocarcinomas Identifies Genetic Alterations That Might Be Targeted With Existing Drugs or Used as Biomarkers^32^ | Singhi AD | 2019 | Not reported. Authors contacted: data not available. |
| 9 | Molecular profiling of patients with pancreatic cancer: initial results from the know your tumor initiative^33^ | Pishvaian MJ | 2018 | Not reported. Authors contacted: not responding. |
| 10 | Statewide Retrospective Review of Familial Pancreatic Cancer in Delaware, and Frequency of Genetic Mutations in Pancreatic Cancer Kindreds^34^ | Catts ZAK | 2018 | Partially reported. Authors not contacted. |
| 11 | Precision medicine for advanced pancreas cancer: The individualizes molecular pancreatic cancer therapy (IMPaCT) Trial^35^ | Chantrill LA | 2015 | Not reported. Authors contacted: not responding. |
| 12 | Clinical implications of genomic alterations in the tumour and circulation of pancreatic cancer patients^36^ | Sausen M | 2015 | Not reported. Authors contacted: data not available. |
| 13 | Whole-exome sequencing of pancreatic cancer defines genetic diversity and therapeutic targets^37^ | Witkiewicz AK | 2015 | Not reported. Authors contacted: not responding. |
| 14 | Germline BRCA Mutations in a Large Clinic-Based Cohort of Patients With Pancreatic Adenocarcinoma^38^ | Holter S | 2015 | Partially reported. Authors not contacted. |
| 15 | The Prevalence of BRCA2 Mutations in Familial Pancreatic Cancer^39^ | Couch F | 2007 | Partially reported. Authors not contacted. |
| 16 | Inherited predisposition to pancreatic adenocarcinoma: role of family history and germ-line p16, BRCA1, and BRCA2 mutations^40^ | Lal G | 2000 | Partially reported. Authors not contacted. |

**Supplementary material 1 references**

1. Page MJ, McKenzie JE, Bossuyt PM, et al: The PRISMA 2020 statement: an updated guideline for reporting systematic reviews. BMJ 372:n71, 2021

2. Hamilton JG, Symecko H, Spielman K, et al: Uptake and acceptability of a mainstreaming model of hereditary cancer multigene panel testing among patients with ovarian, pancreatic, and prostate cancer. Genet Med 23:2105-2113, 2021

3. Lincoln SE, Nussbaum RL, Kurian AW, et al: Yield and Utility of Germline Testing Following Tumor Sequencing in Patients With Cancer. JAMA Netw Open 3:e2019452, 2020

4. Pishvaian MJ, Blais EM, Brody JR, et al: Overall survival in patients with pancreatic cancer receiving matched therapies following molecular profiling: a retrospective analysis of the Know Your Tumor registry trial. Lancet Oncol 21:508-518, 2020

5. Lamping M, Benary M, Leyvraz S, et al: Support of a molecular tumour board by an evidence-based decision management system for precision oncology. Eur J Cancer 127:41-51, 2020

6. Pishvaian MJ, Blais EM, Brody JR, et al: Outcomes in Patients With Pancreatic Adenocarcinoma With Genetic Mutations in DNA Damage Response Pathways: Results From the Know Your Tumor Program. JCO Precision Oncology:1-10, 2019

7. Sicklick JK, Kato S, Okamura R, et al: Molecular profiling of cancer patients enables personalized combination therapy: the I-PREDICT study. Nat Med 25:744-750, 2019

8. Unseld M, Mader R, Baumann L, et al: Feasibility of personalized treatment concepts in gastrointestinal malignancies: Sub-group results of prospective clinical phase II trial EXACT. Chin J Cancer Res 30:508-515, 2018

9. Mitri ZI, Parmar S, Johnson B, et al: Implementing a comprehensive translational oncology platform: from molecular testing to actionability. J Transl Med 16:358, 2018

10. Basse C, Morel C, Alt M, et al: Relevance of a molecular tumour board (MTB) for patients' enrolment in clinical trials: experience of the Institut Curie. ESMO Open 3:e000339, 2018

11. Cancer Genome Atlas Research Network. Electronic address aadhe, Cancer Genome Atlas Research N: Integrated Genomic Characterization of Pancreatic Ductal Adenocarcinoma. Cancer Cell 32:185-203 e13, 2017

12. Radovich M, Kiel PJ, Nance SM, et al: Clinical benefit of a precision medicine based approach for guiding treatment of refractory cancers. Oncotarget 7:56491-56500, 2016

13. Borecka M, Zemankova P, Vocka M, et al: Mutation analysis of the PALB2 gene in unselected pancreatic cancer patients in the Czech Republic. Cancer Genet 209:199-204, 2016

14. Dalton WB, Forde PM, Kang H, et al: Personalized Medicine in the Oncology Clinic: Implementation and Outcomes of the Johns Hopkins Molecular Tumor Board. JCO Precis Oncol 2017, 2017

15. Waddell N, Pajic M, Patch AM, et al: Whole genomes redefine the mutational landscape of pancreatic cancer. Nature 518:495-501, 2015

16. Le Tourneau C, Delord JP, Goncalves A, et al: Molecularly targeted therapy based on tumour molecular profiling versus conventional therapy for advanced cancer (SHIVA): a multicentre, open-label, proof-of-concept, randomised, controlled phase 2 trial. Lancet Oncol 16:1324-34, 2015

17. Salo-Mullen EE, O'Reilly EM, Kelsen DP, et al: Identification of germline genetic mutations in patients with pancreatic cancer. Cancer 121:4382-8, 2015

18. Grant RC, Selander I, Connor AA, et al: Prevalence of germline mutations in cancer predisposition genes in patients with pancreatic cancer. Gastroenterology 148:556-64, 2015

19. Tsimberidou AM, Wen S, Hong DS, et al: Personalized medicine for patients with advanced cancer in the phase I program at MD Anderson: validation and landmark analyses. Clin Cancer Res 20:4827-36, 2014

20. Lucas AL, Shakya R, Lipsyc MD, et al: High prevalence of BRCA1 and BRCA2 germline mutations with loss of heterozygosity in a series of resected pancreatic adenocarcinoma and other neoplastic lesions. Clin Cancer Res 19:3396-403, 2013

21. Harinck F, Kluijt I, van Mil SE, et al: Routine testing for PALB2 mutations in familial pancreatic cancer families and breast cancer families with pancreatic cancer is not indicated. Eur J Hum Genet 20:577-9, 2012

22. Von Hoff DD, Stephenson JJ, Jr., Rosen P, et al: Pilot study using molecular profiling of patients' tumors to find potential targets and select treatments for their refractory cancers. J Clin Oncol 28:4877-83, 2010

23. Ferrone CR, Levine DA, Tang LH, et al: BRCA germline mutations in Jewish patients with pancreatic adenocarcinoma. J Clin Oncol 27:433-8, 2009

24. Figer A, Irmin L, Geva R, et al: The rate of the 6174delT founder Jewish mutation in BRCA2 in patients with non-colonic gastrointestinal tract tumours in Israel. Br J Cancer 84:478-81, 2001

25. Wang Y, Golesworthy B, Cuggia A, et al: Oncology clinic-based germline genetic testing for exocrine pancreatic cancer enables timely return of results and unveils low uptake of cascade testing. J Med Genet 59:793-800, 2022

26. Momtaz P, O'Connor CA, Chou JF, et al: Pancreas cancer and BRCA: A critical subset of patients with improving therapeutic outcomes. Cancer 127:4393-4402, 2021

27. Peretti U, Cavaliere A, Niger M, et al: Germinal BRCA1-2 pathogenic variants (gBRCA1-2pv) and pancreatic cancer: epidemiology of an Italian patient cohort. ESMO Open 6:100032, 2021

28. Lowder CY, Dhir T, Goetz AB, et al: A step towards personalizing next line therapy for resected pancreatic and related cancer patients: A single institution's experience. Surg Oncol 33:118-125, 2020

29. Hu H, Zhu Y, Pu N, et al: Association of Germline Variants in Human DNA Damage Repair Genes and Response to Adjuvant Chemotherapy in Resected Pancreatic Ductal Adenocarcinoma. J Am Coll Surg 231:527-535 e14, 2020

30. Yadav S, Kasi PM, Bamlet WR, et al: Effect of Germline Mutations in Homologous Recombination Repair Genes on Overall Survival of Patients with Pancreatic Adenocarcinoma. Clin Cancer Res 26:6505-6512, 2020

31. Schwartz M, Korenbaum C, Benfoda M, et al: Familial pancreatic adenocarcinoma: A retrospective analysis of germline genetic testing in a French multicentre cohort. Clin Genet 96:579-584, 2019

32. Singhi AD, George B, Greenbowe JR, et al: Real-Time Targeted Genome Profile Analysis of Pancreatic Ductal Adenocarcinomas Identifies Genetic Alterations That Might Be Targeted With Existing Drugs or Used as Biomarkers. Gastroenterology 156:2242-2253 e4, 2019

33. Pishvaian MJ, Bender RJ, Halverson D, et al: Molecular Profiling of Patients with Pancreatic Cancer: Initial Results from the Know Your Tumor Initiative. Clin Cancer Res 24:5018-5027, 2018

34. Catts ZA, Baig MK, Milewski B, et al: Statewide Retrospective Review of Familial Pancreatic Cancer in Delaware, and Frequency of Genetic Mutations in Pancreatic Cancer Kindreds. Ann Surg Oncol 23:1729-35, 2016

35. Chantrill LA, Nagrial AM, Watson C, et al: Precision Medicine for Advanced Pancreas Cancer: The Individualized Molecular Pancreatic Cancer Therapy (IMPaCT) Trial. Clin Cancer Res 21:2029-37, 2015

36. Sausen M, Phallen J, Adleff V, et al: Clinical implications of genomic alterations in the tumour and circulation of pancreatic cancer patients. Nat Commun 6:7686, 2015

37. Witkiewicz AK, McMillan EA, Balaji U, et al: Whole-exome sequencing of pancreatic cancer defines genetic diversity and therapeutic targets. Nat Commun 6:6744, 2015

38. Holter S, Borgida A, Dodd A, et al: Germline BRCA Mutations in a Large Clinic-Based Cohort of Patients With Pancreatic Adenocarcinoma. J Clin Oncol 33:3124-9, 2015

39. Couch FJ, Johnson MR, Rabe KG, et al: The prevalence of BRCA2 mutations in familial pancreatic cancer. Cancer Epidemiol Biomarkers Prev 16:342-6, 2007

40. Lal G, Liu G, Schmocker B, et al: Inherited predisposition to pancreatic adenocarcinoma: role of family history and germ-line p16, BRCA1, and BRCA2 mutations. Cancer Res 60:409-16, 2000
