# Supplementary material for: A systematic review and meta-analysis of germline BRCA mutations in pancreatic cancer patients identifies global and racial disparities in access to genetic testing
Source: ESMO Open. 2023 Feb 21;8(2):100881. doi: 10.1016/j.esmoop.2023.100881 (PMC10163165; doi:10.1016/j.esmoop.2023.100881)

Metanalysis results

Index Pg.

1. White patients 4

*1.1 Tested 4*

1.1.1 Funnel plot 4

1.1.2 Metanalysis 4

1.1.3 Temporal analysis 5

*1.2 Positive for any BRCA 5*

1.2.1 Funnel plot 5

1.2.2 Metanalysis 6

1.2.3 Temporal analysis 7

*1.3 Positive for BRCA1 7*

1.3.1 Funnel plot 7

1.3.2 Metanalysis 8

1.3.3 Temporal analysis 8

*1.4 Positive for BRCA2 9*

1.4.1 Funnel plot 9

1.4.2 Metanalysis 9

1.4.3 Temporal analysis 10

2. African American patients 11

*2.1 Tested 11*

2.1.1 Funnel plot 11

2.1.2 Metanalysis 11

2.1.3 Temporal analysis 12

*2.2 Positive for any BRCA 12*

2.2.1 Funnel plot 12

2.2.2 Metanalysis 13

2.2.3 Temporal analysis 13

*2.3 Positive for BRCA1 13*

2.3.1 Funnel plot 13

2.3.2 Metanalysis 14

2.3.3 Temporal analysis 14

*2.4 Positive for BRCA2 14*

2.4.1 Funnel plot 14

2.4.2 Metanalysis 15

2.4.3 Temporal analysis 15

3. Asian patients 16

*3.1 Tested 16*

3.1.1 Funnel plot 16

3.1.2 Metanalysis 16

3.1.3 Temporal analysis 17

3.2 *Positive for any BRCA* 17

3.2.1 Funnel plot 17

3.2.2 Metanalysis 18

3.2.3 Temporal analysis 18

3.3 *Positive for BRCA1* 18

3.3.1 Funnel plot 18

3.3.2 Metanalysis 19

3.3.3 Temporal analysis 19

3.4 *Positive for BRCA2* 19

3.4.1 Funnel plot 19

3.4.2 Metanalysis 20

3.4.3 Temporal analysis 20

4. Hispanic patients 21

*4.1 Tested 21*

4.1.1 Funnel plot 21

4.1.2 Metanalysis 21

4.1.3 Temporal analysis 22

4.2 *Positive for any BRCA* 22

4.2.1 Funnel plot 22

4.2.2 Metanalysis 23

4.2.3 Temporal analysis 23

_____________________________________________________________________

1. White patients

*1.1 Tested*

1.1.1 Funnel plot


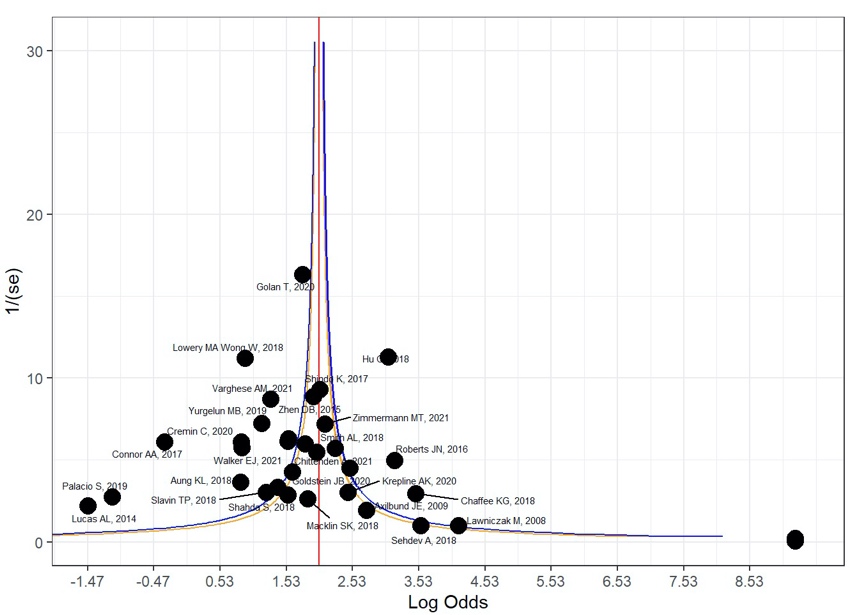


1.1.2 Metanalysis


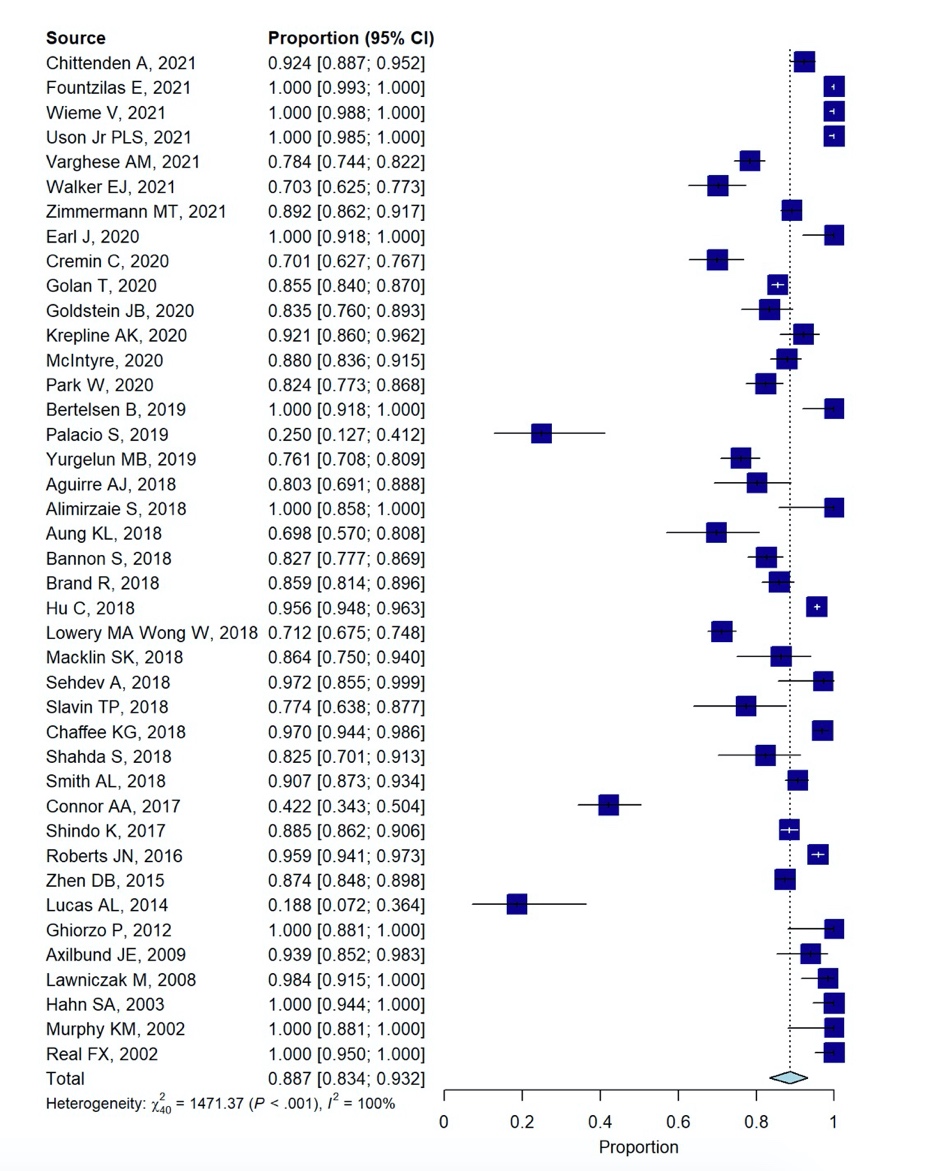


1.1.3 Temporal analysis

*
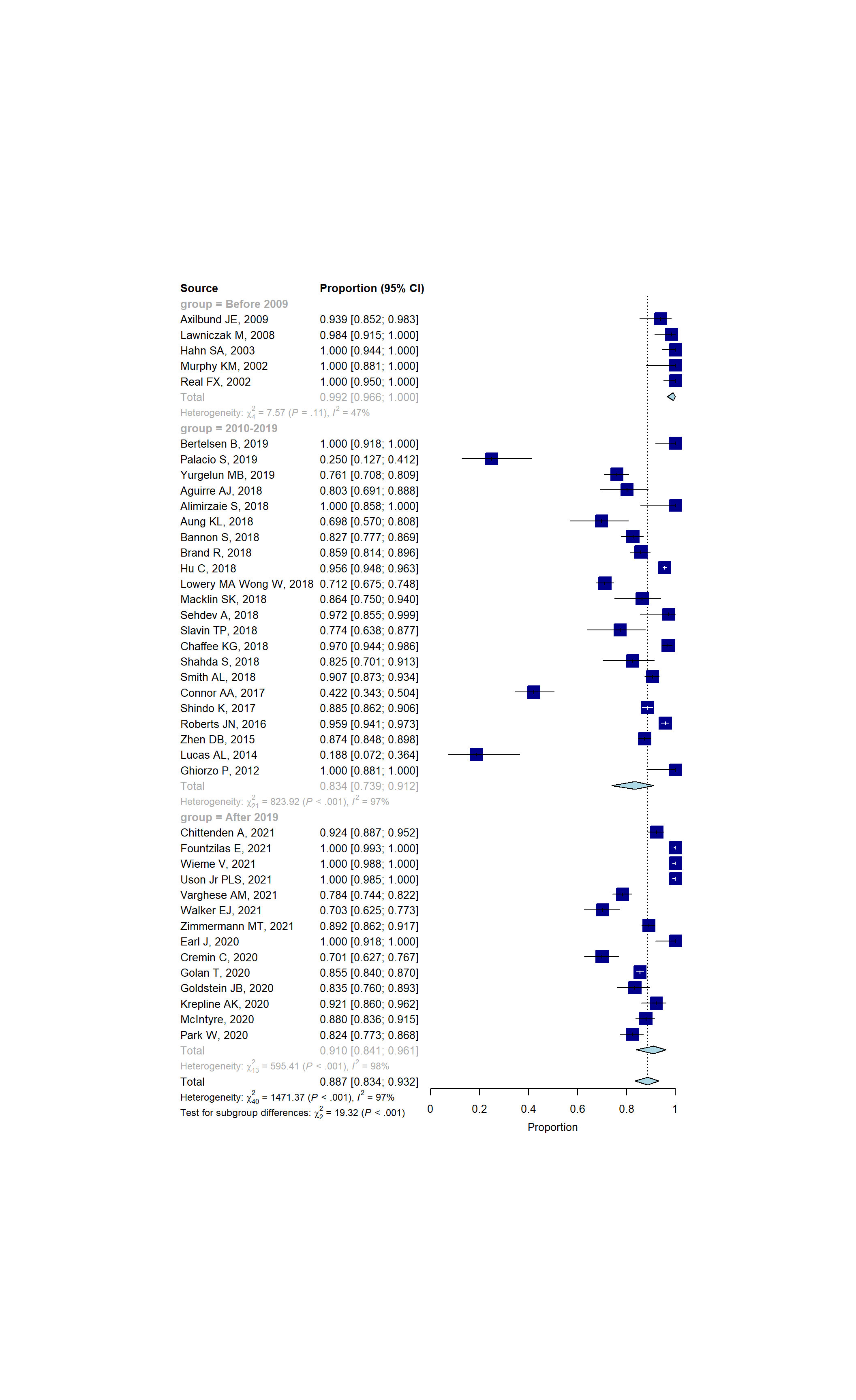
*

*1.2 Positive for any BRCA*

1.2.1 Funnel plot


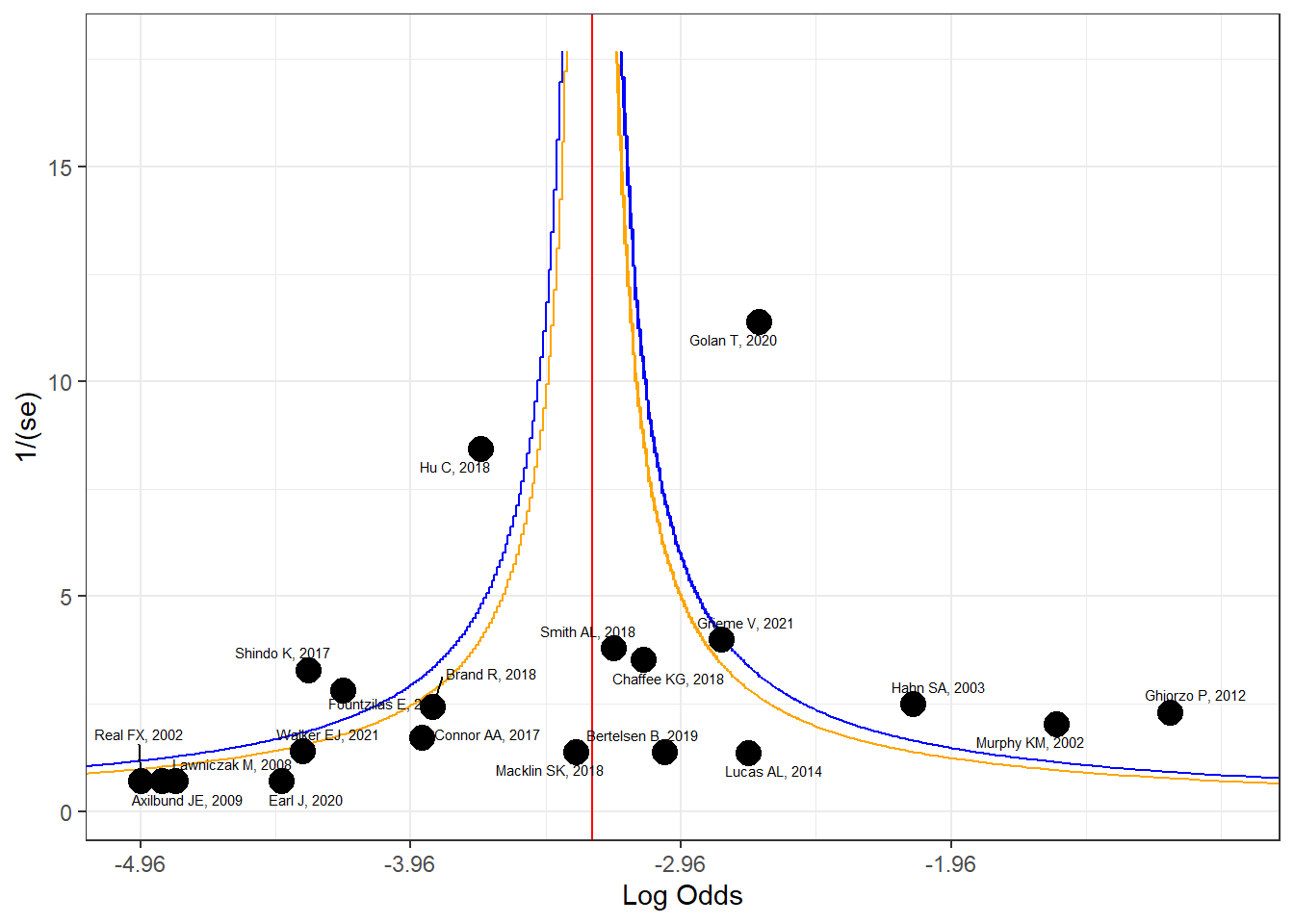


1.2.2 Metanalysis


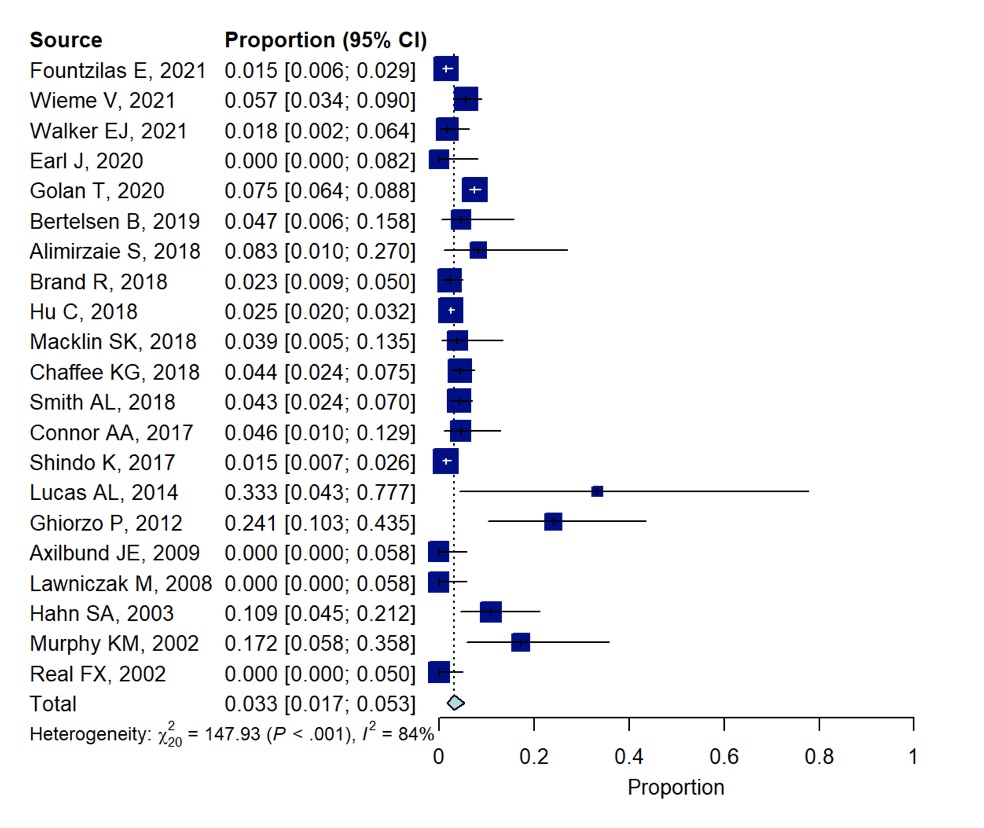


*1.2.3 Temporal analysis*

*
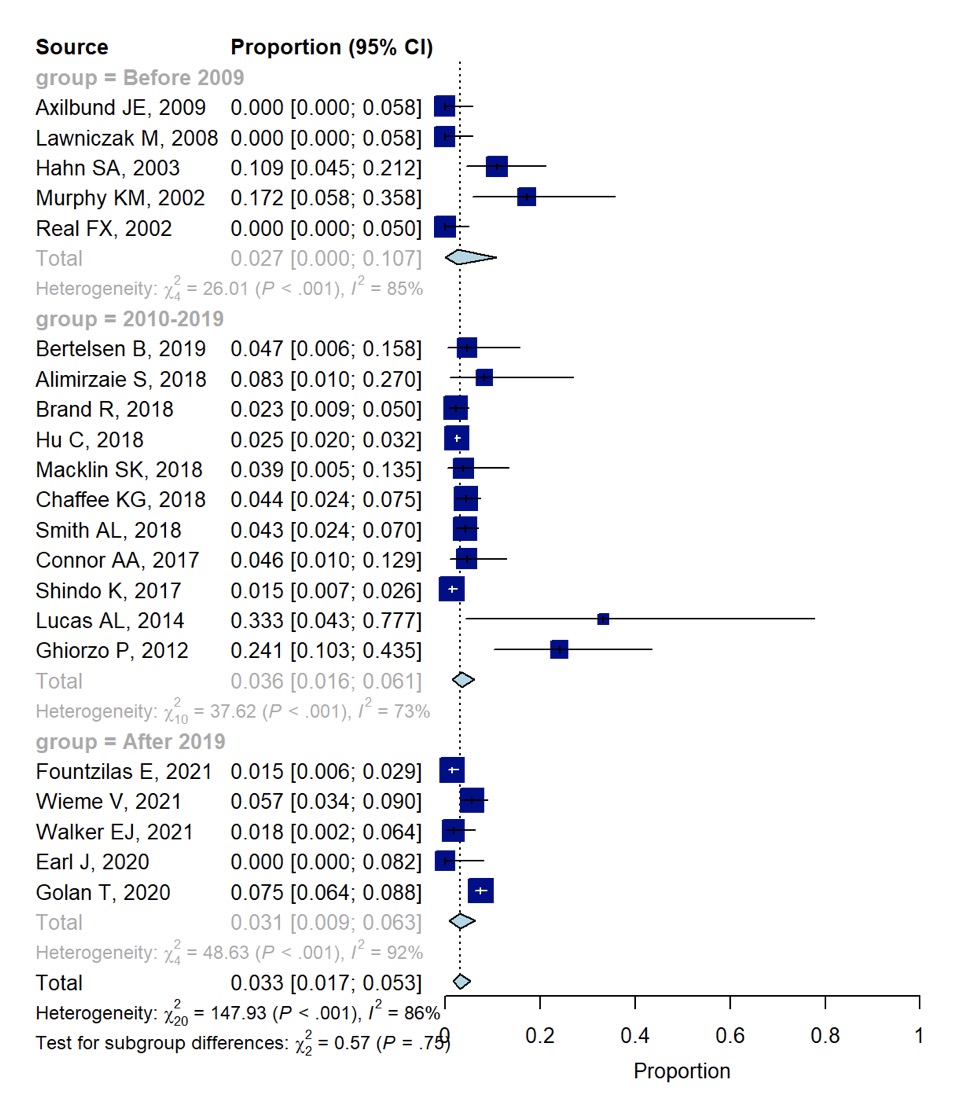
*

*1.3 Positive for BRCA1*

1.3.1 Funnel plot


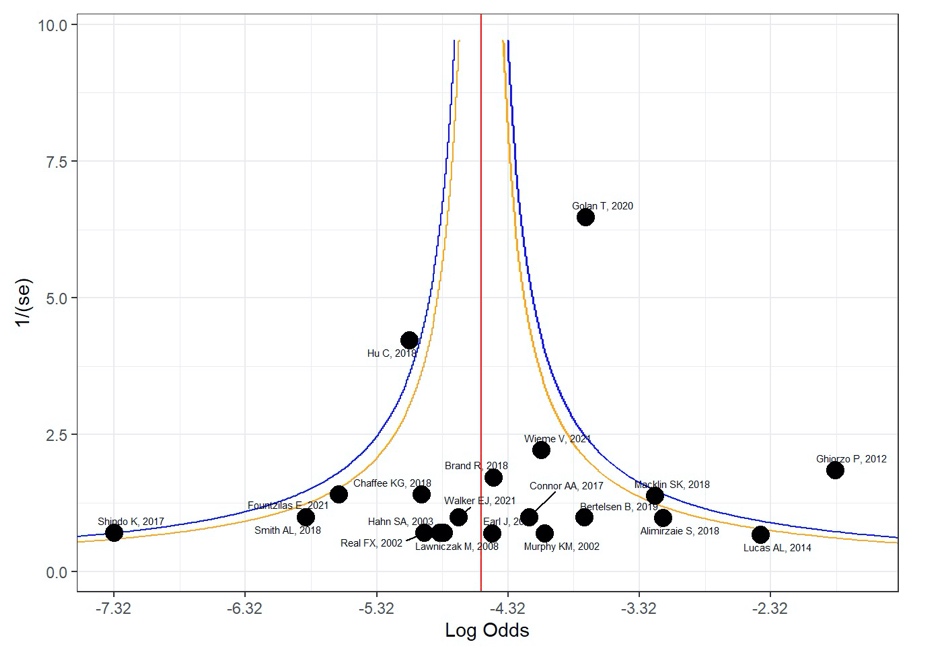


1.3.2 Metanalysis


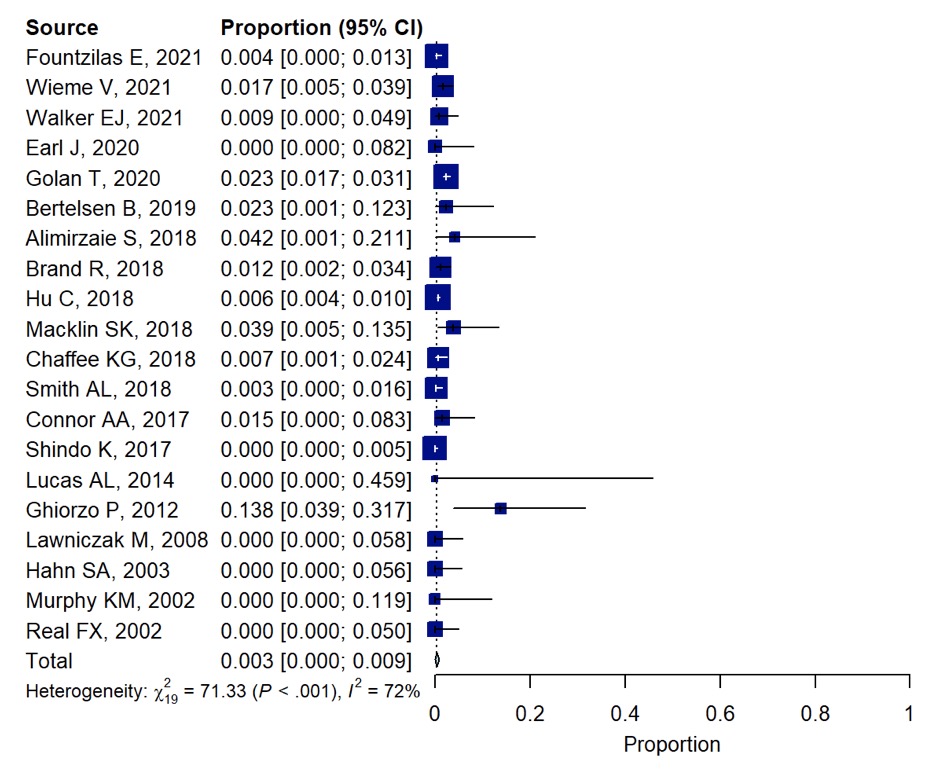


1.3.3 Temporal analysis


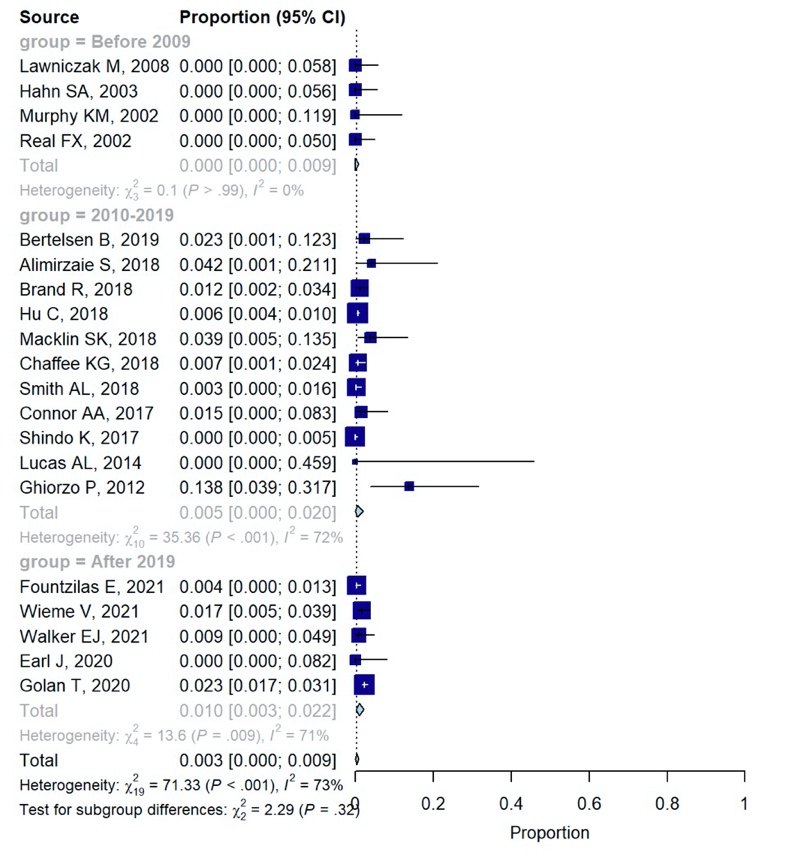


*1.4 Positive for BRCA2*

1.4.1 Funnel plot


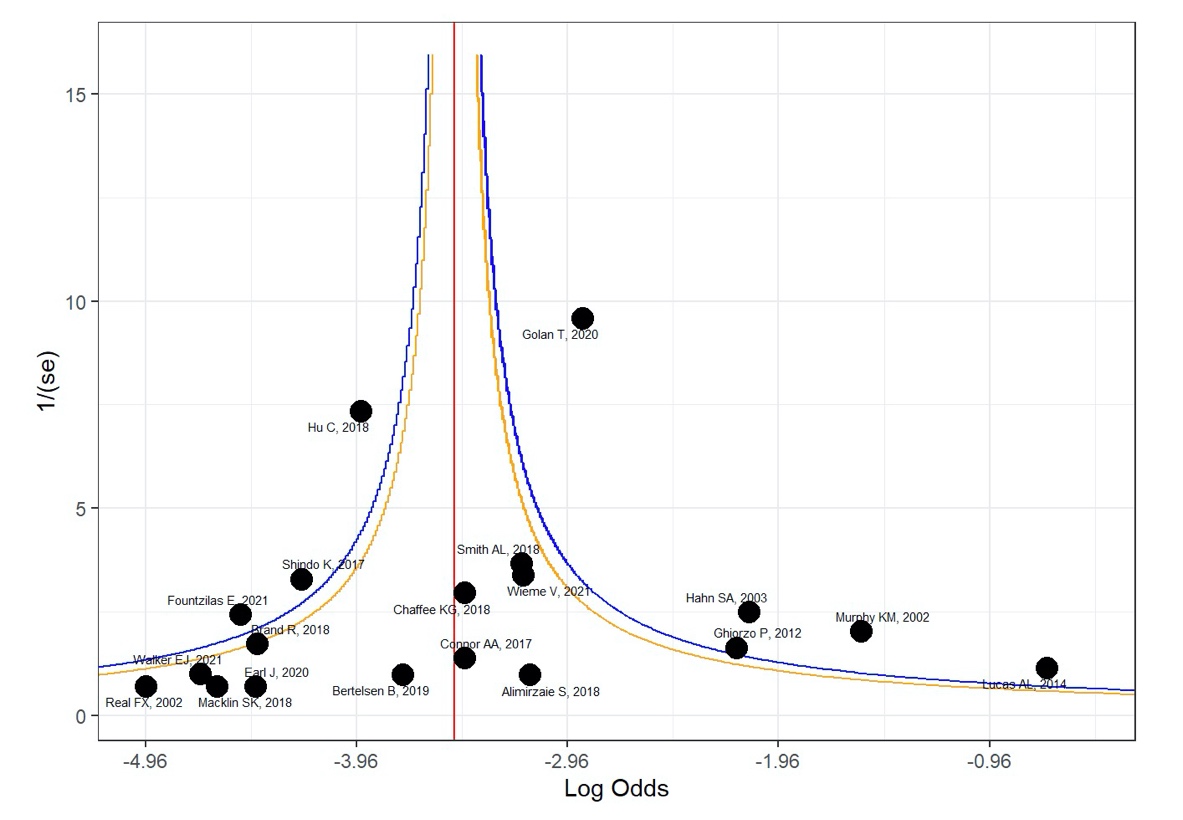


1.4.2 Metanalysis


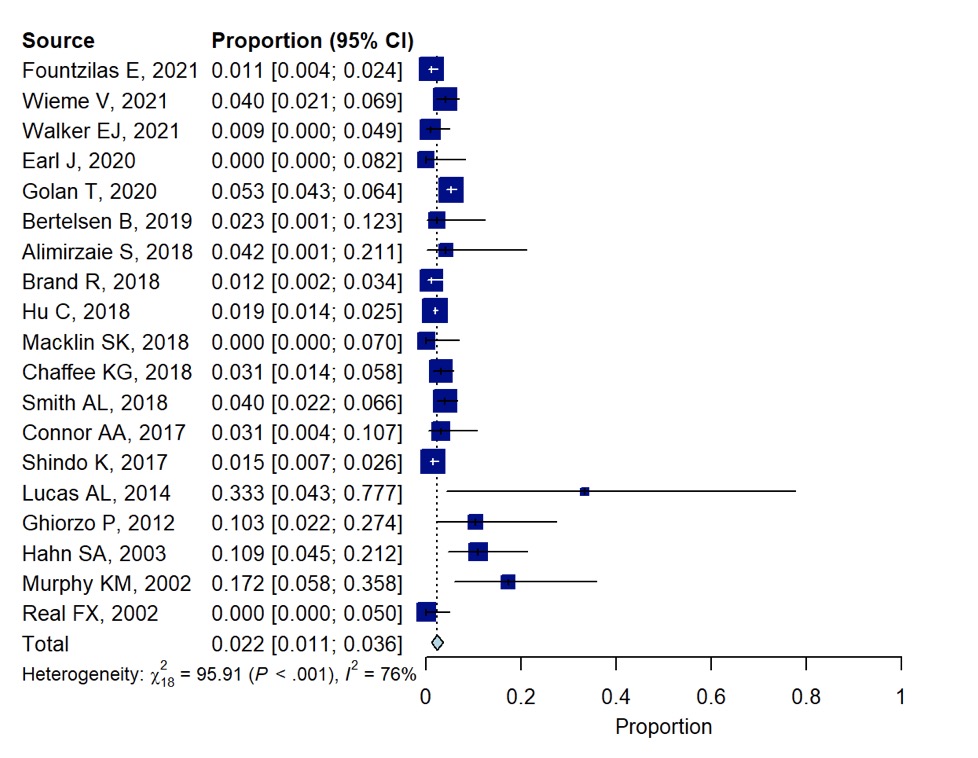


1.4.3 Temporal analysis


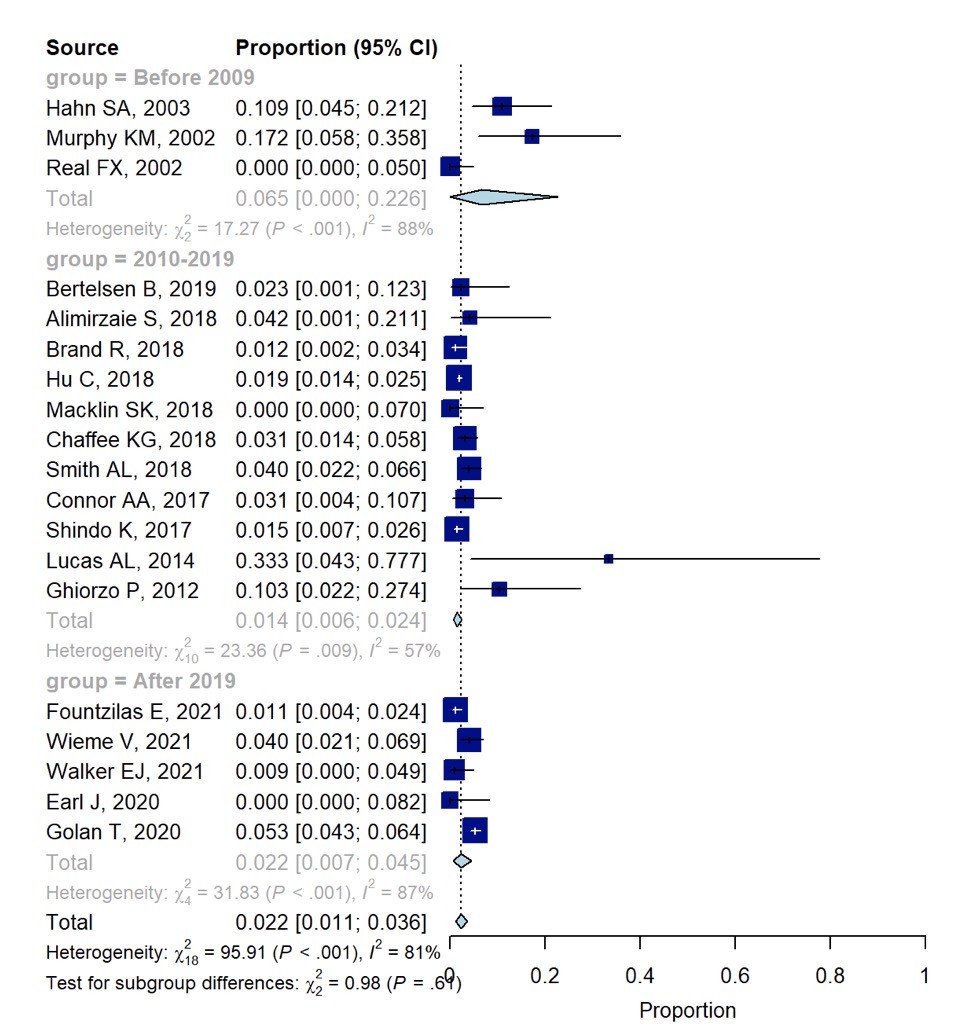


2. African American patients

*2.1 Tested*

2.1.1 Funnel plot
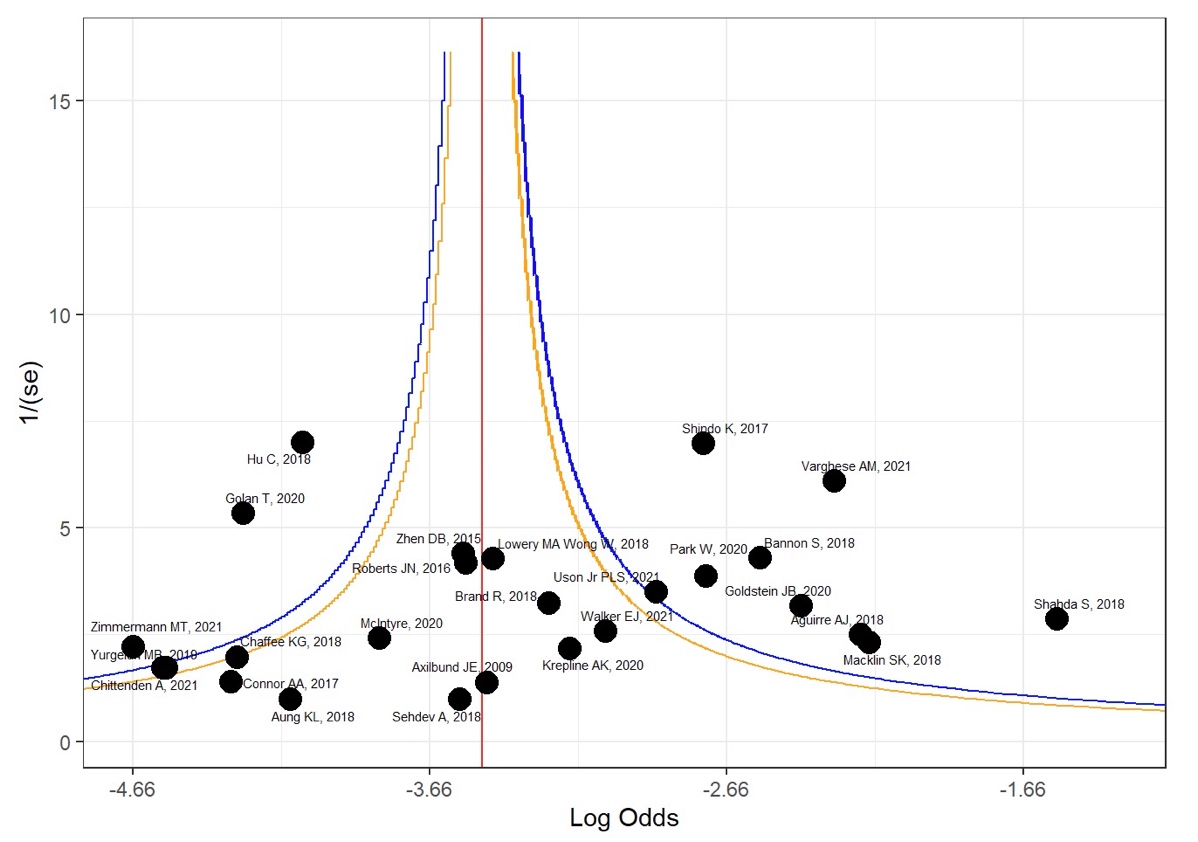


2.1.2 Metanalysis


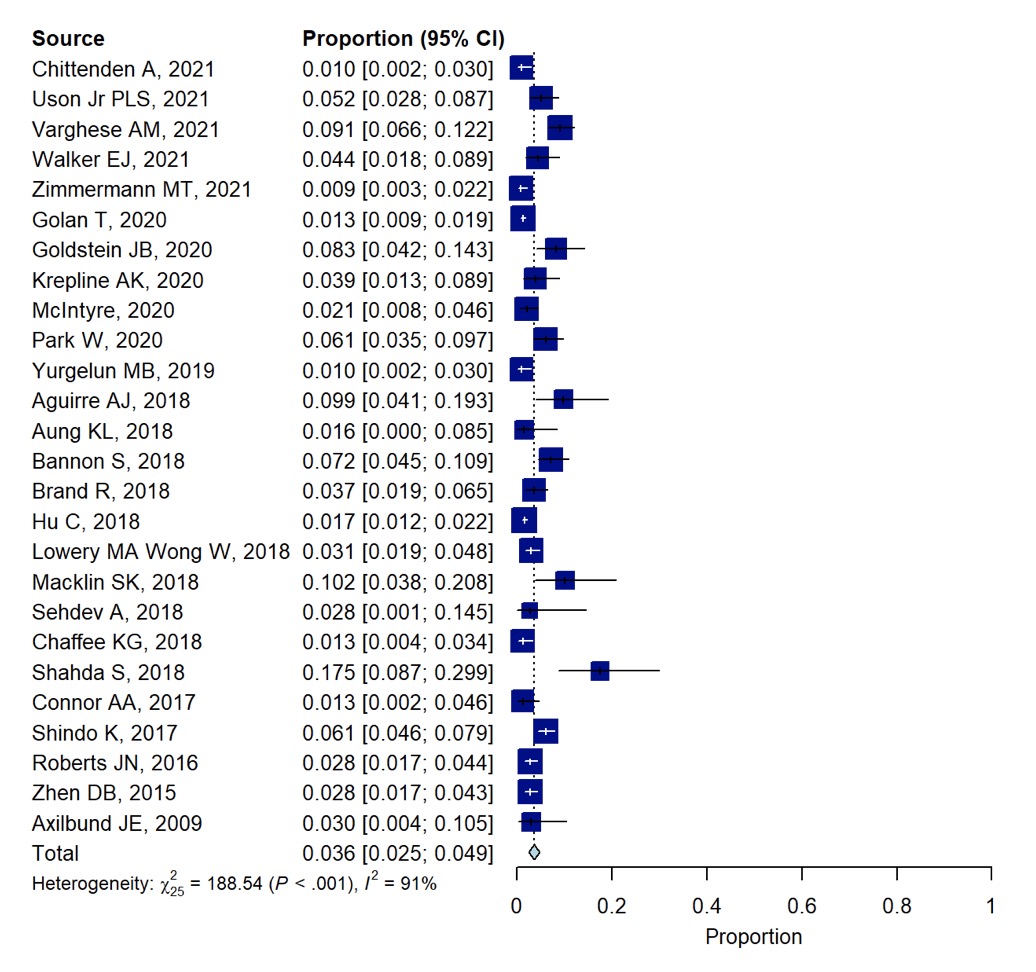


2.1.3 Temporal analysis

*
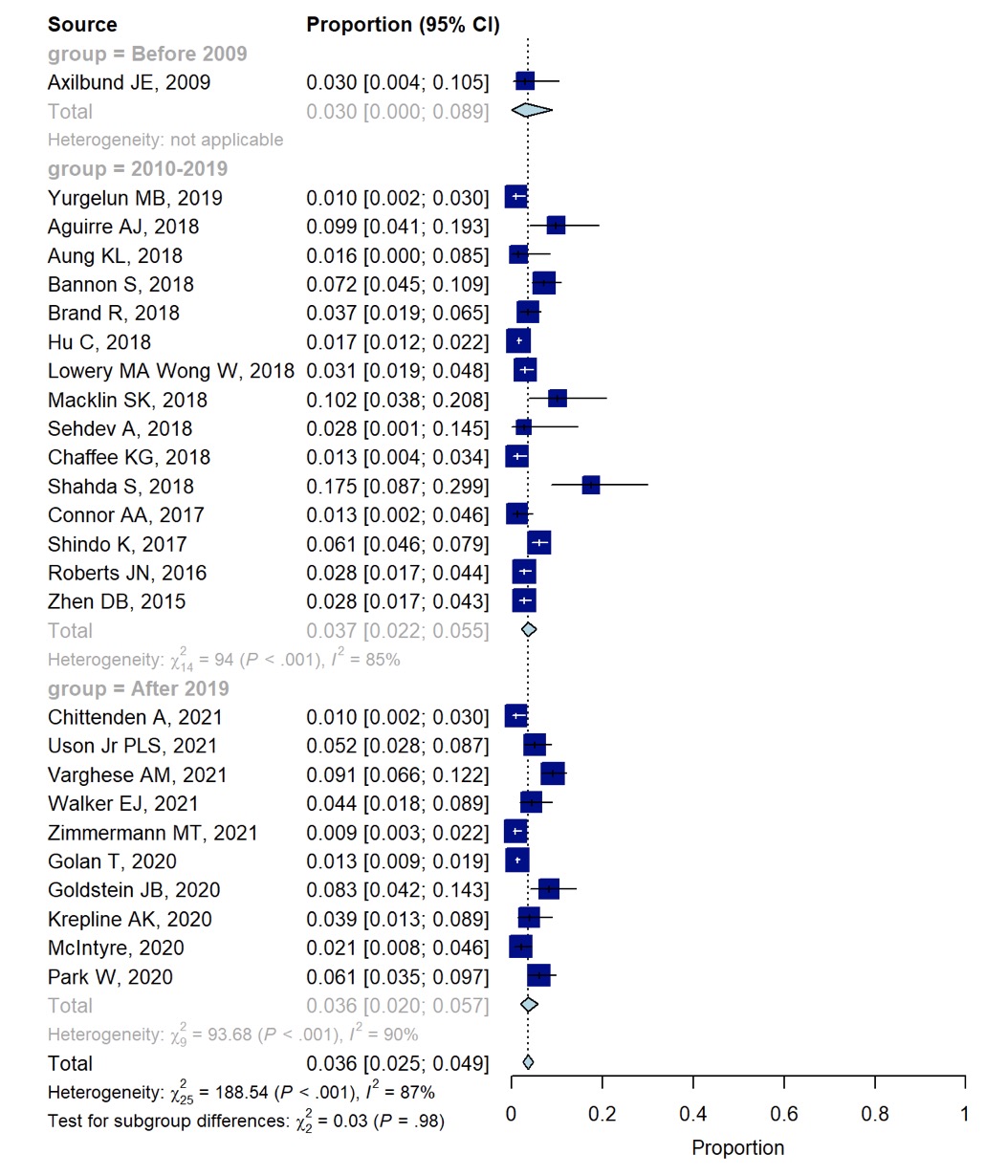
*

*2.2 Positive for any BRCA*

2.2.1 Funnel plot


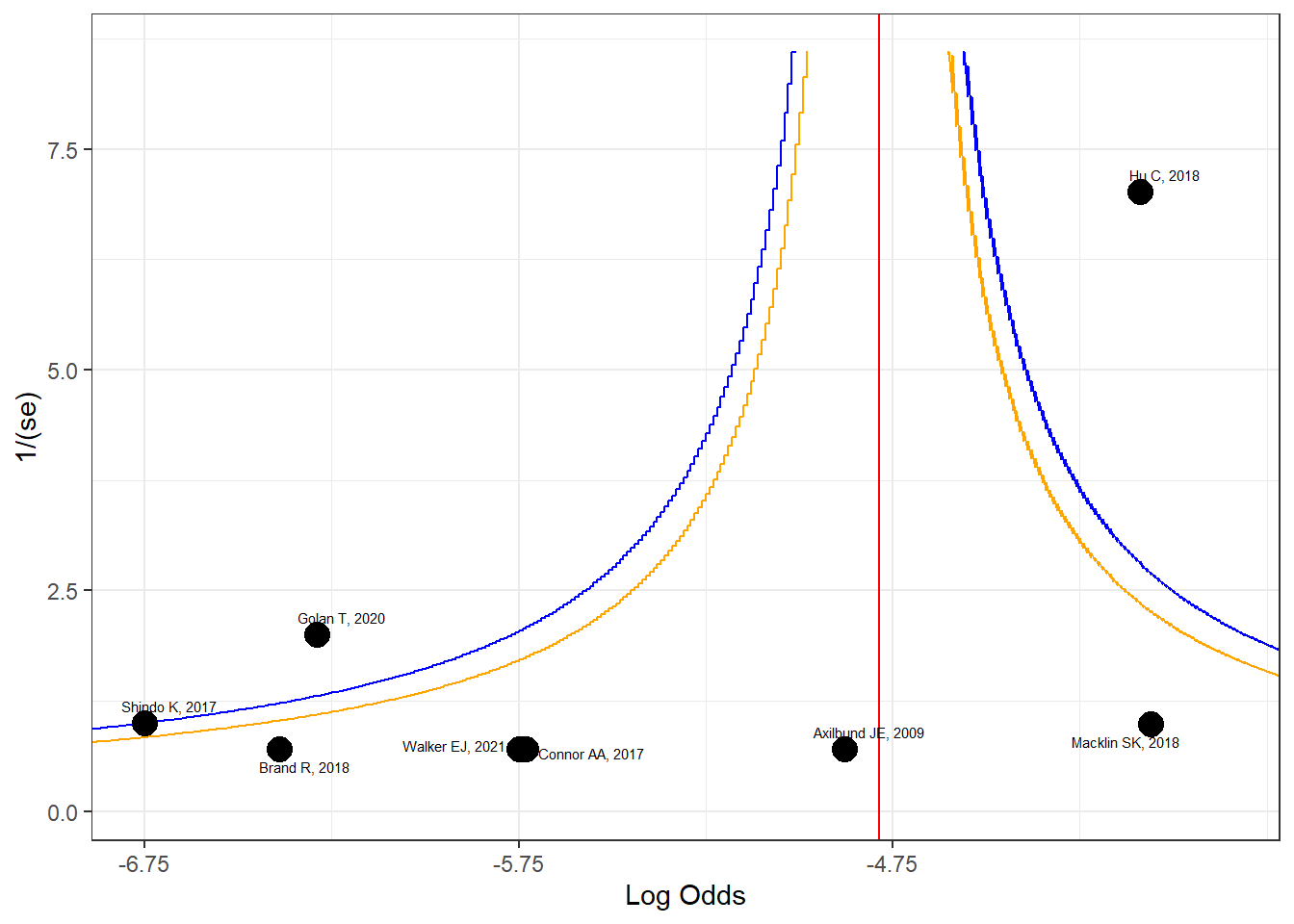


2.2.2 Metanalysis


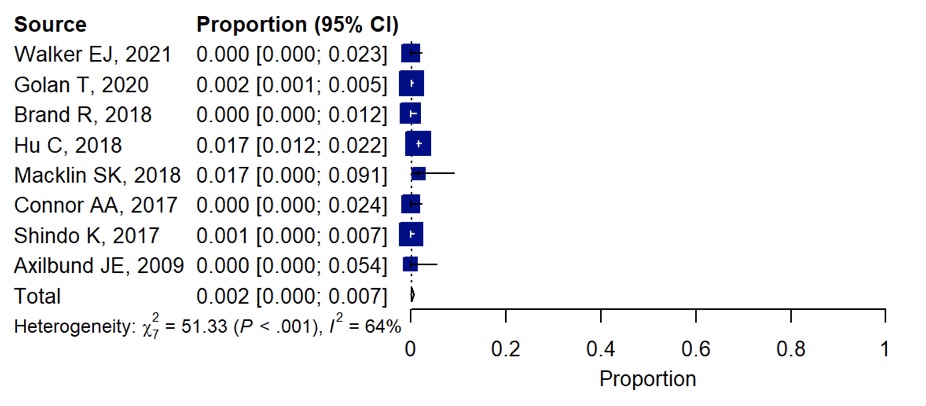


2.2.3 Temporal analysis


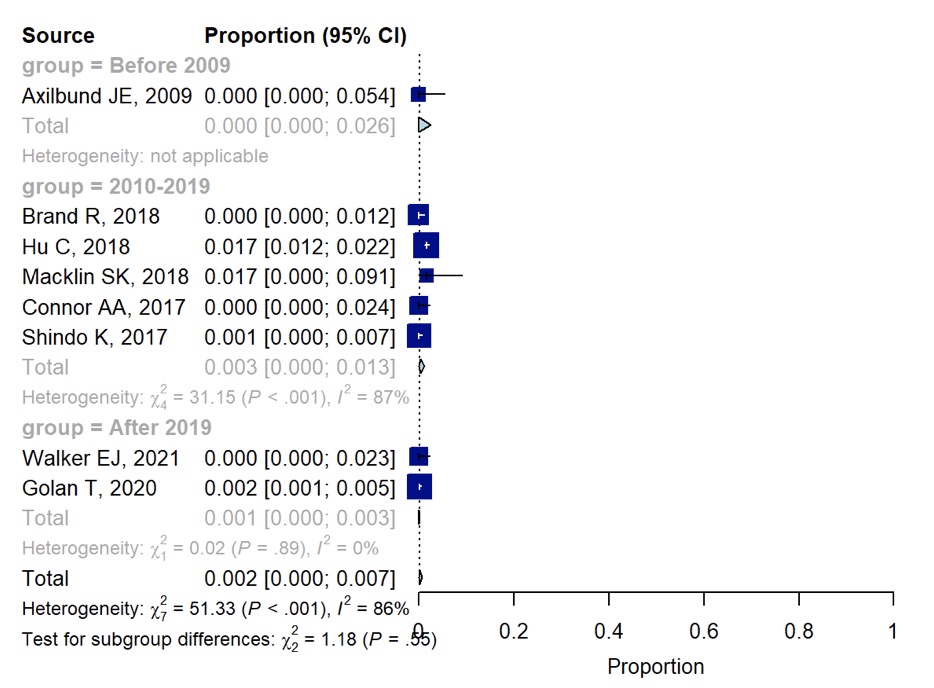


*2.3 Positive for BRCA1*

2.3.1 Funnel plot


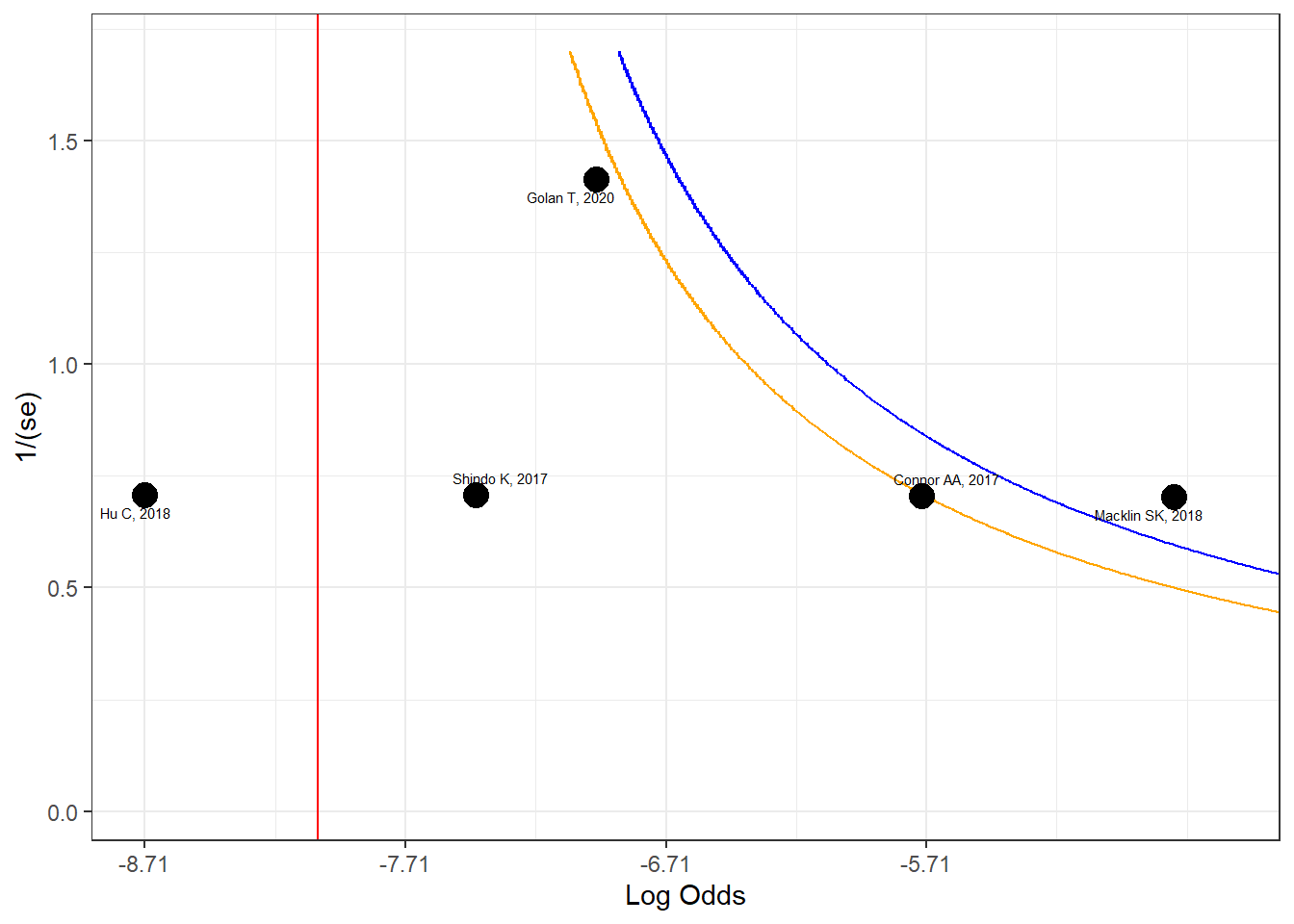


2.3.2 Metanalysis


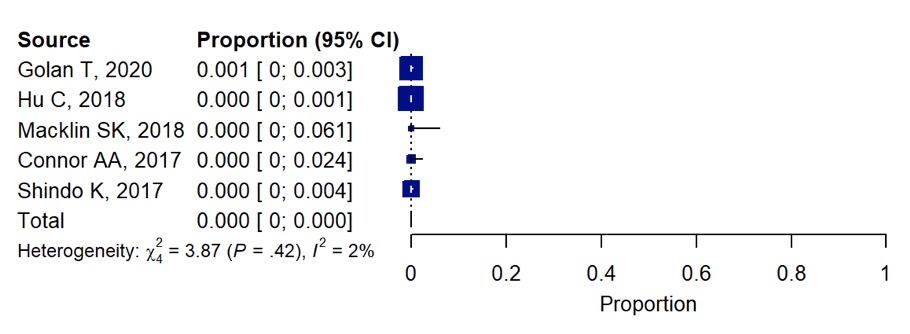


2.3.3 Temporal analysis

*
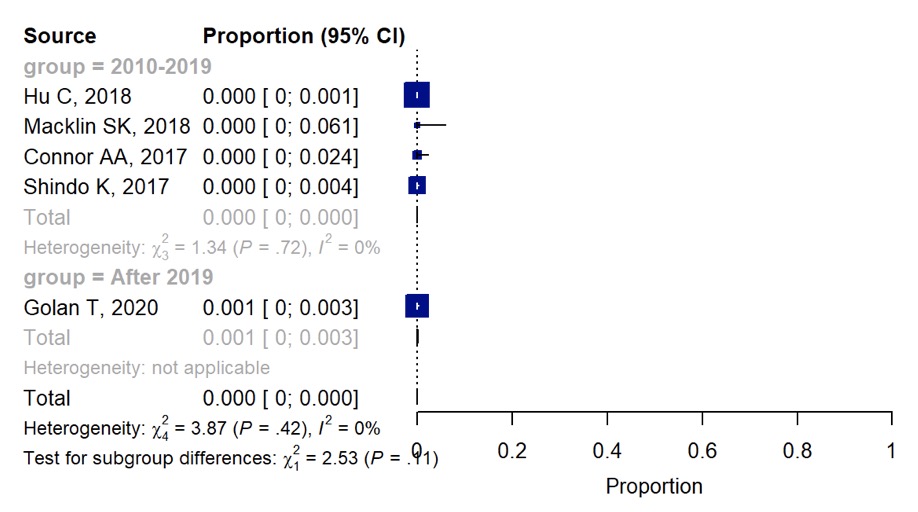
*

*2.4 Positive for BRCA2*

2.4.1 Funnel plot


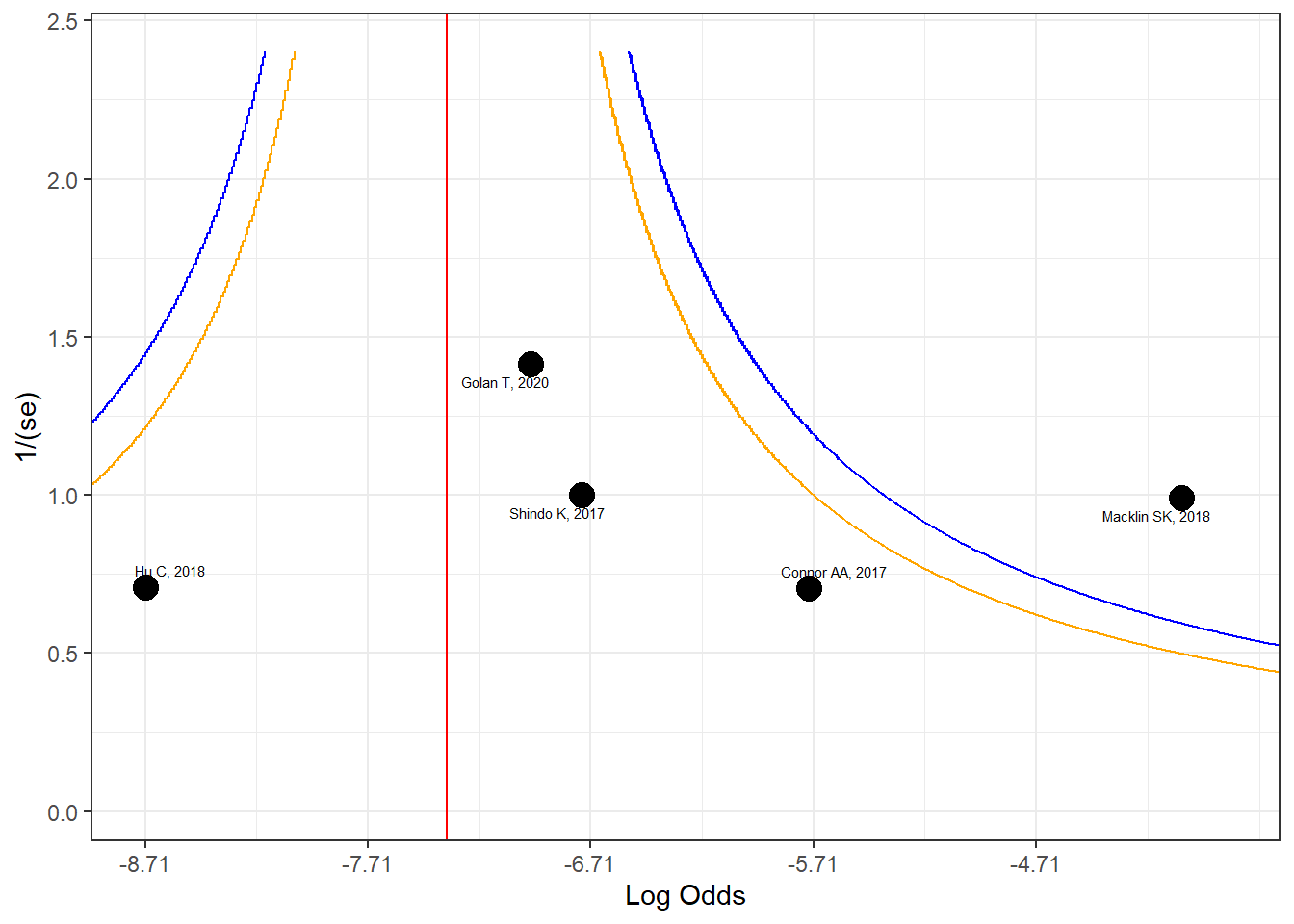


2.4.2 Metanalysis


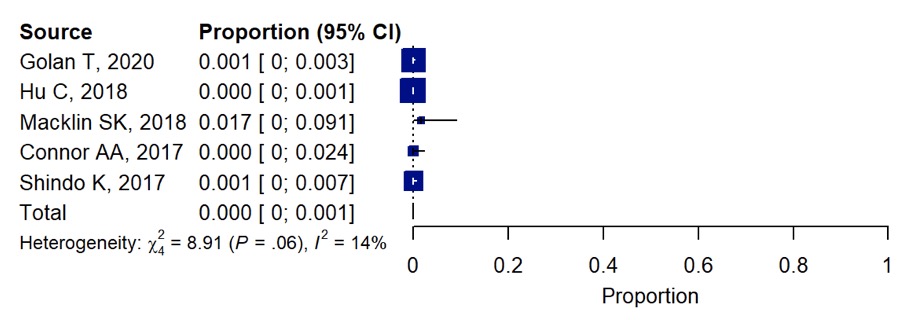


2.4.3 Temporal analysis


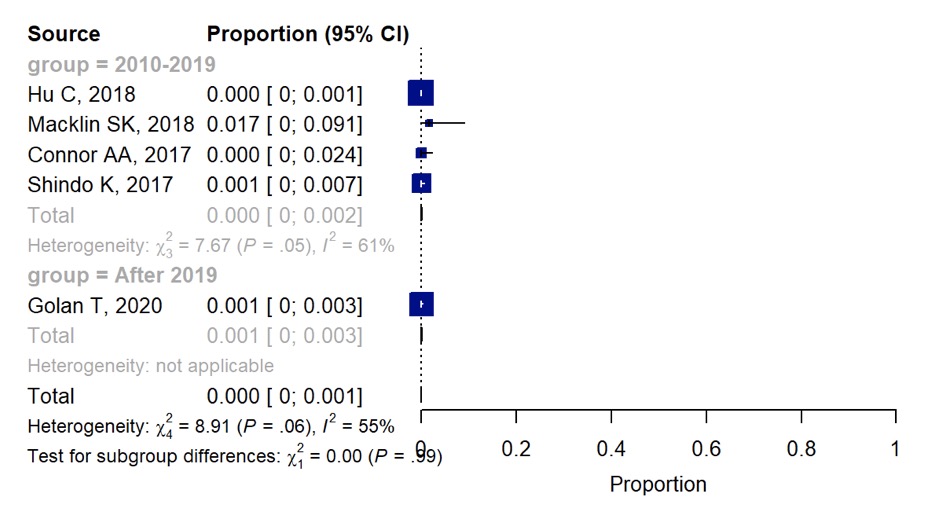


3. Asian patients

*3.1 Tested*

3.1.1 Funnel plot


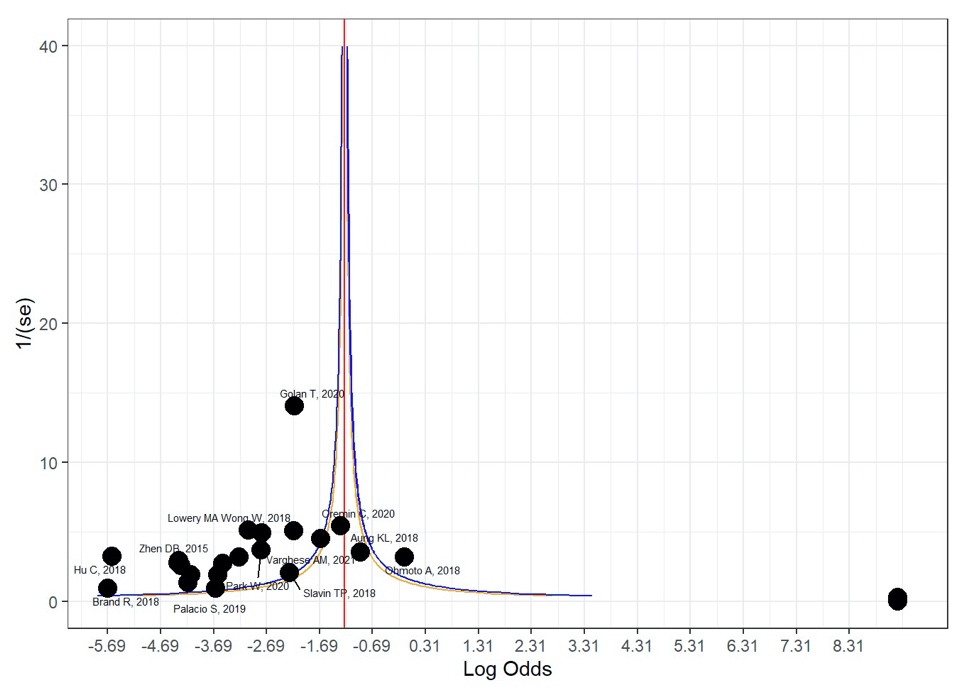


3.1.2 Metanalysis


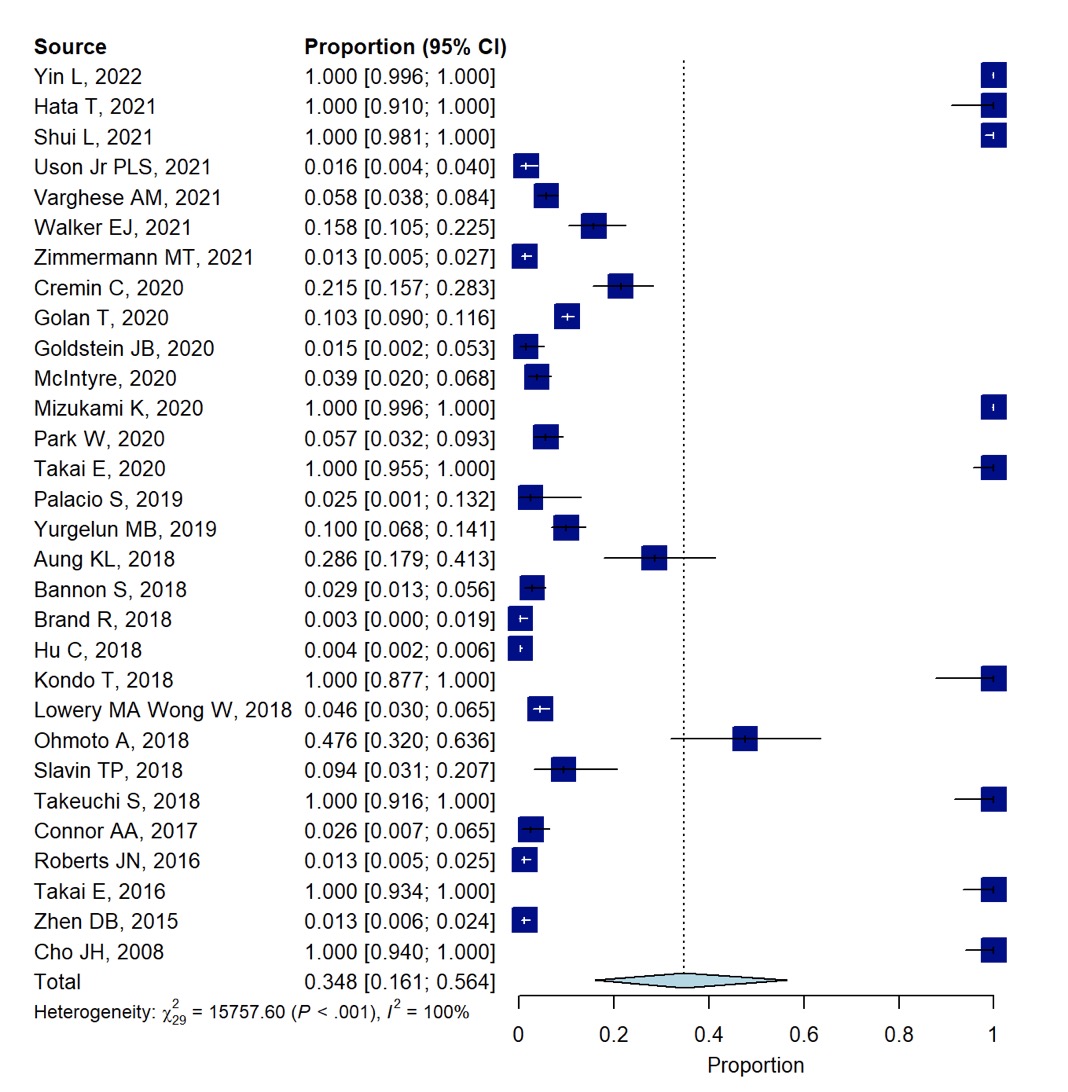


3.1.3 Temporal analysis


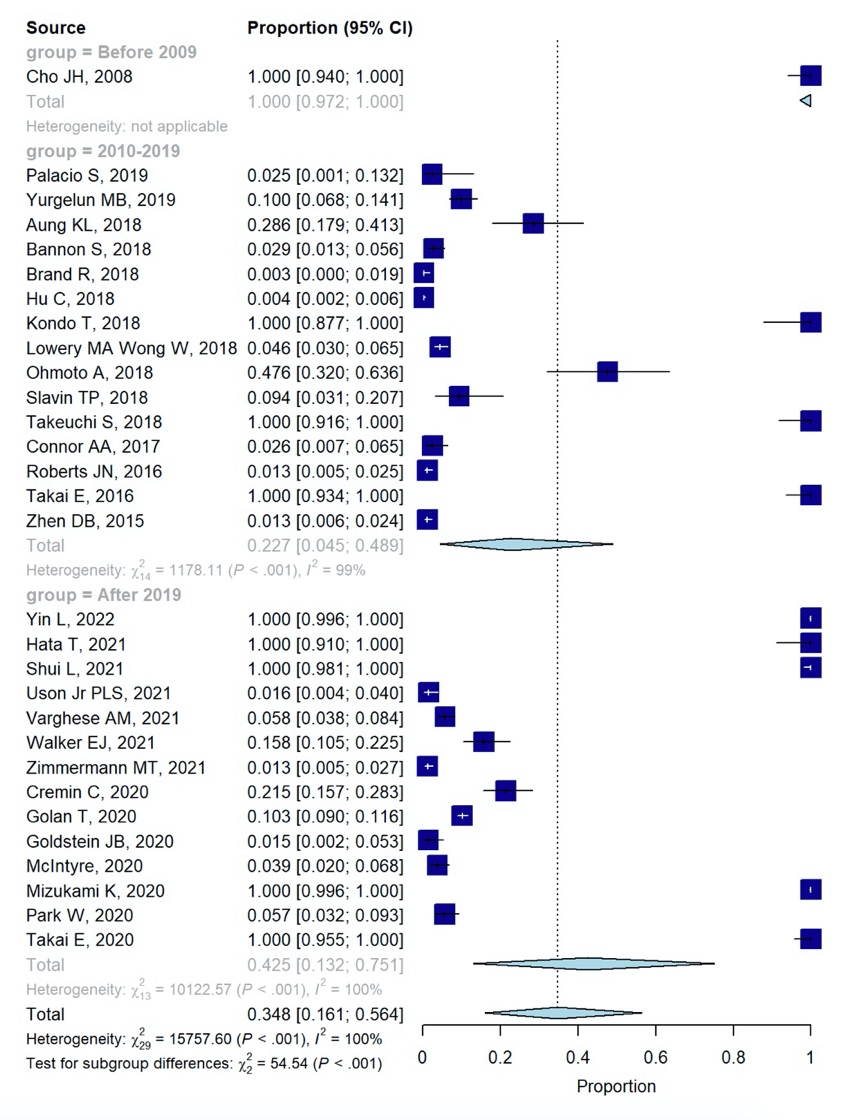


*3.2 Positive for any BRCA*

3.2.1 Funnel plot


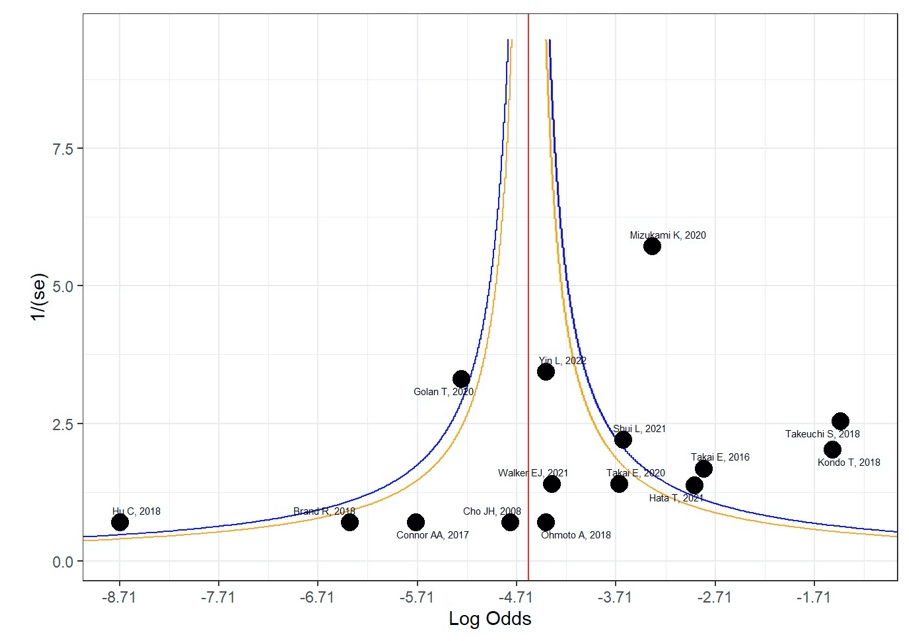


3.2.2 Metanalysis


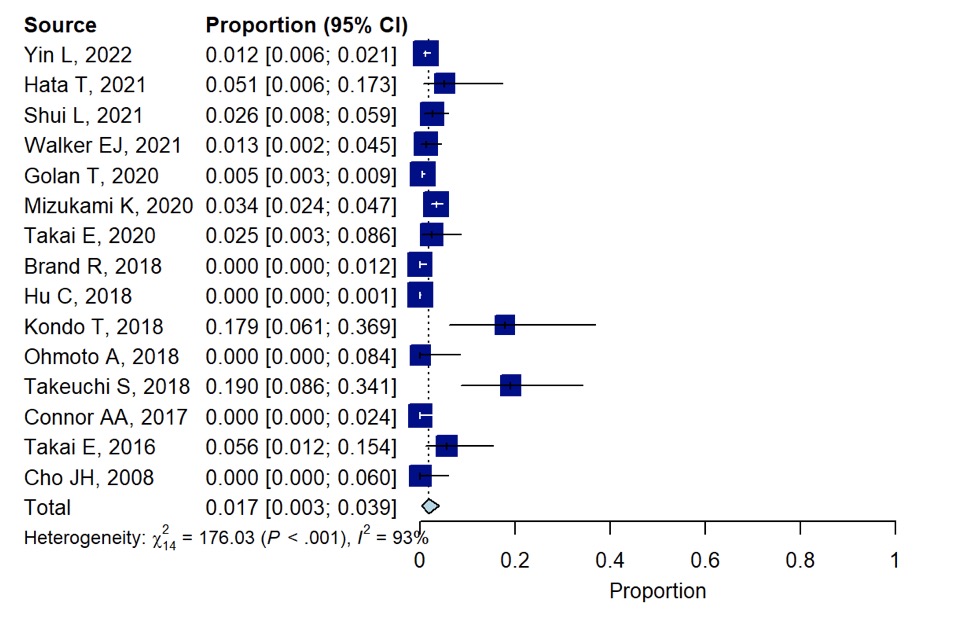


3.2.3 Temporal analysis


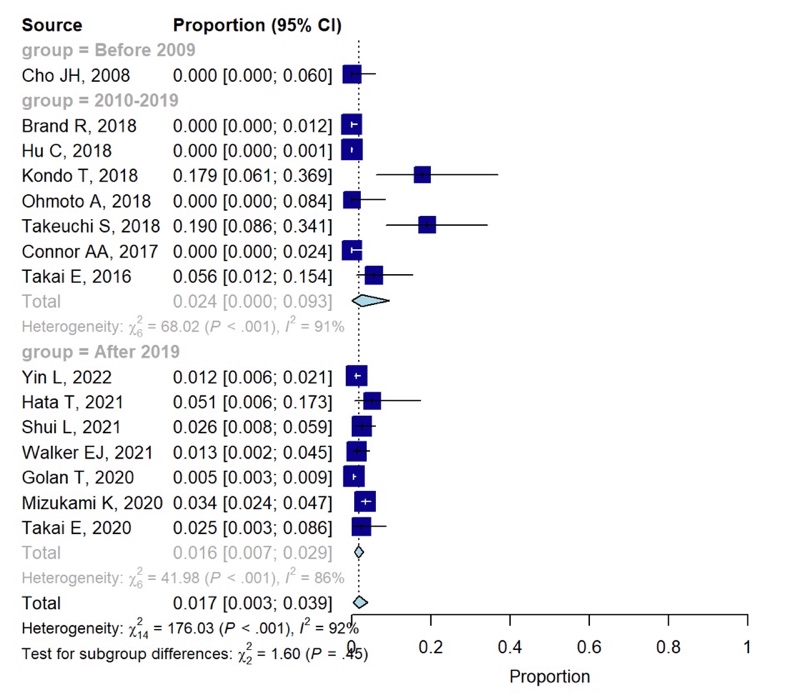


*3.3 Positive for BRCA1*

3.3.1 Funnel plot


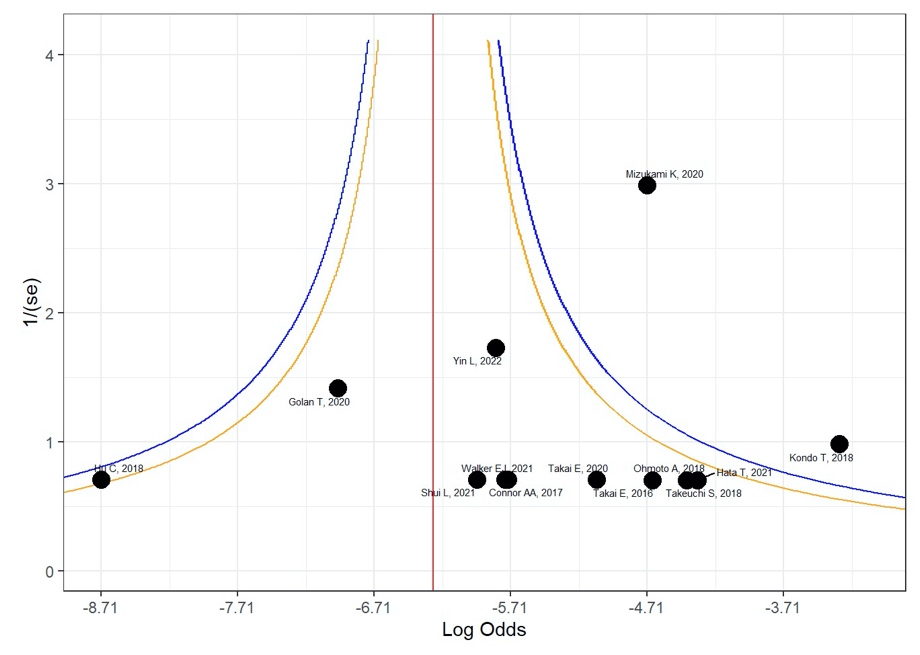


3.3.2 Metanalysis


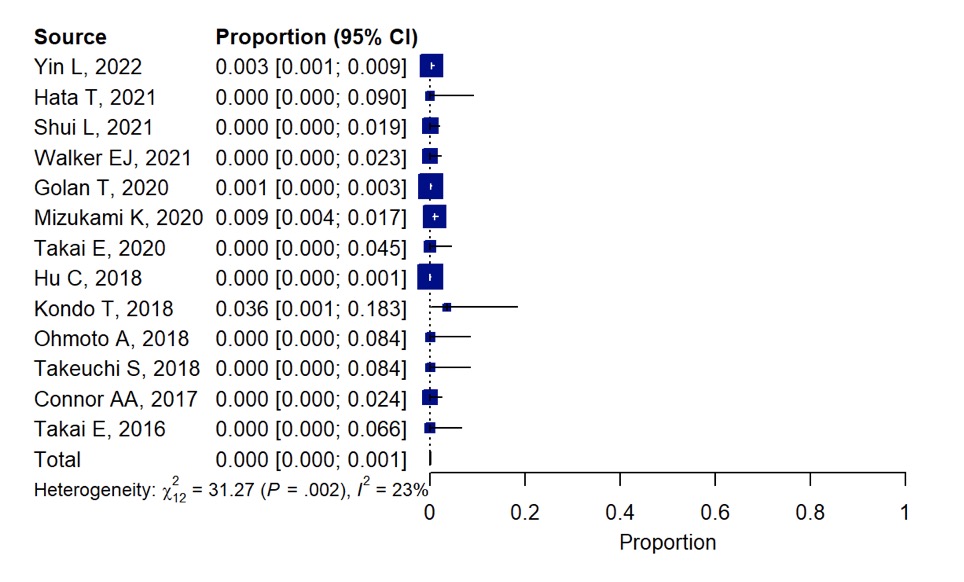


3.3.3 Temporal analysis


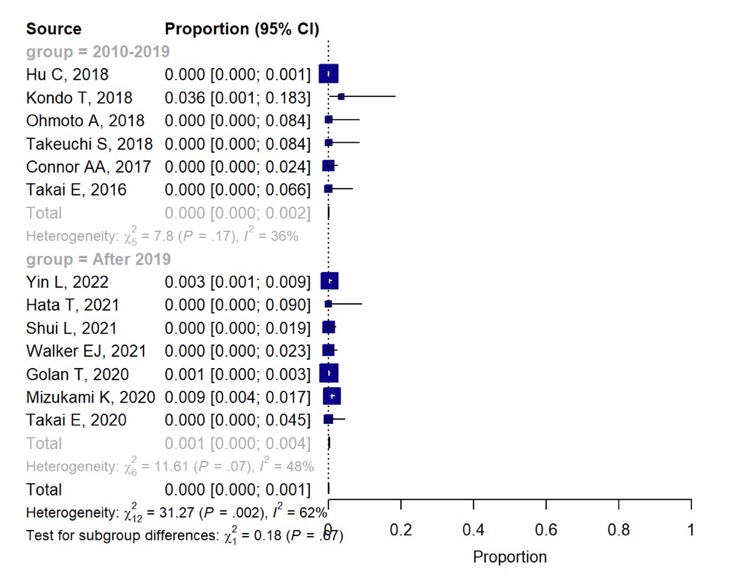


*3.4 Positive for BRCA2*

3.4.1 Funnel plot


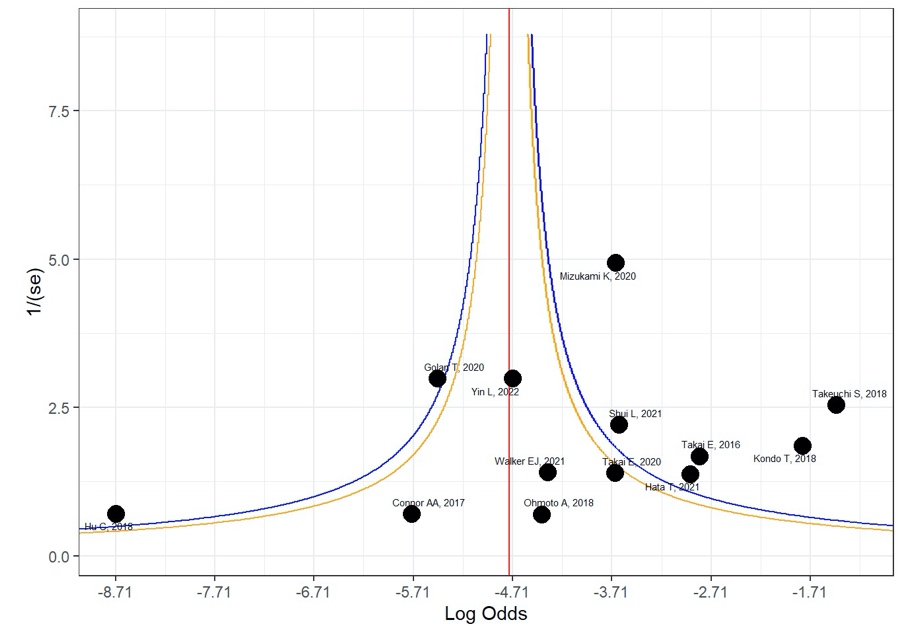


3.4.2 Metanalysis


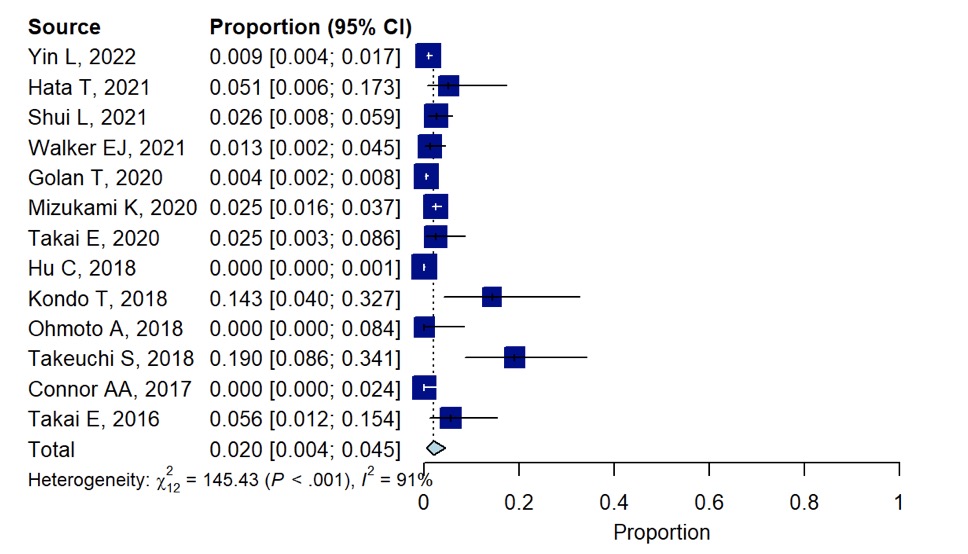


3.4.3 Temporal analysis


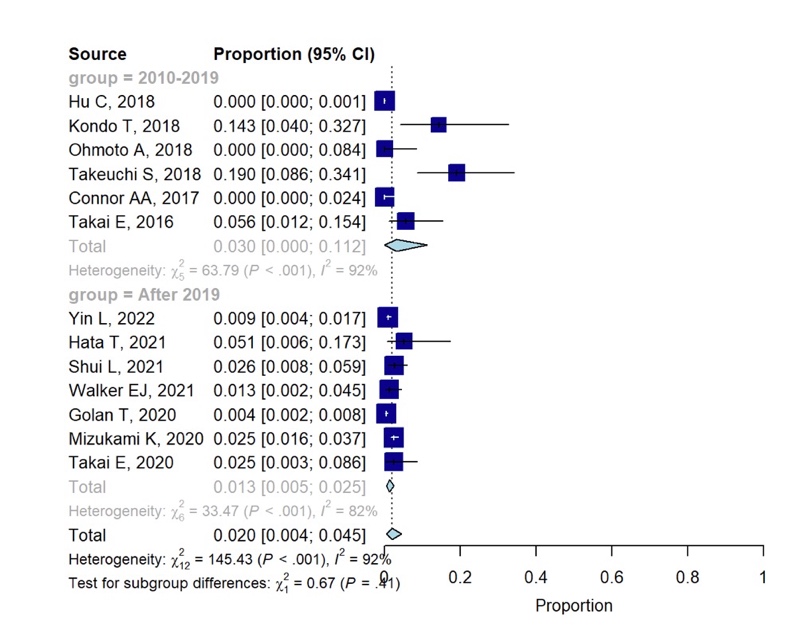


4. Hispanic patients

*4.1 Tested*

4.1.1 Funnel plot


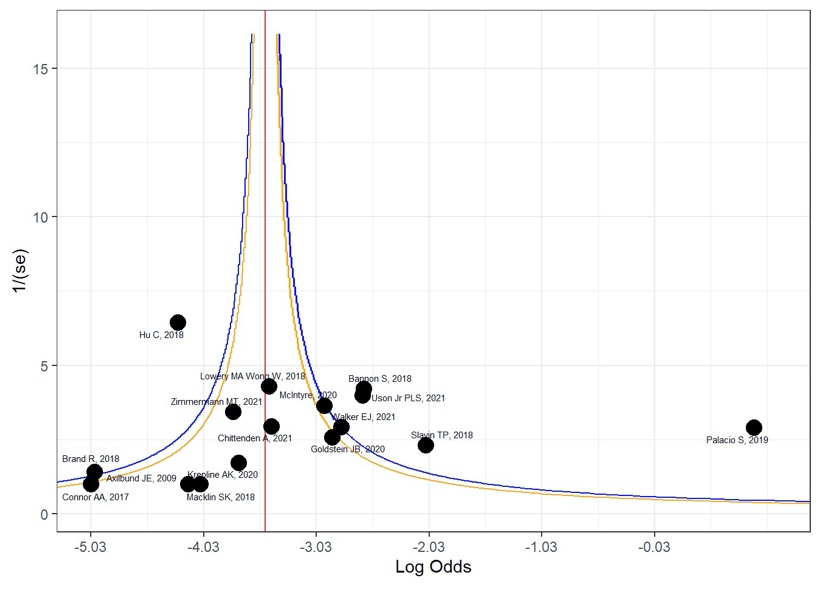


4.1.2 Metanalysis


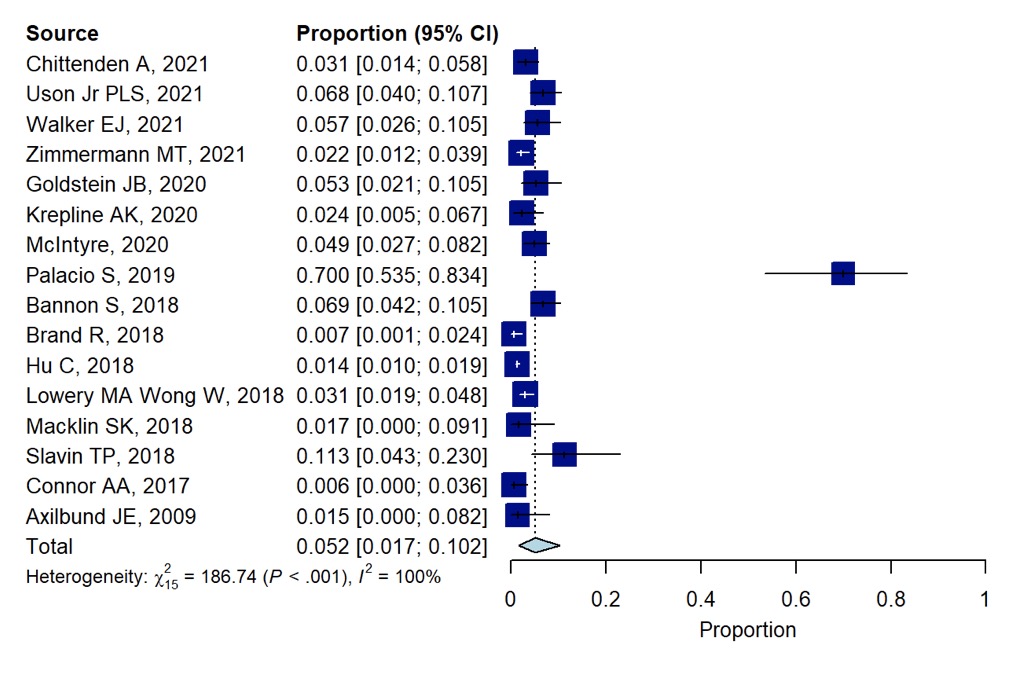


4.1.3 Temporal analysis


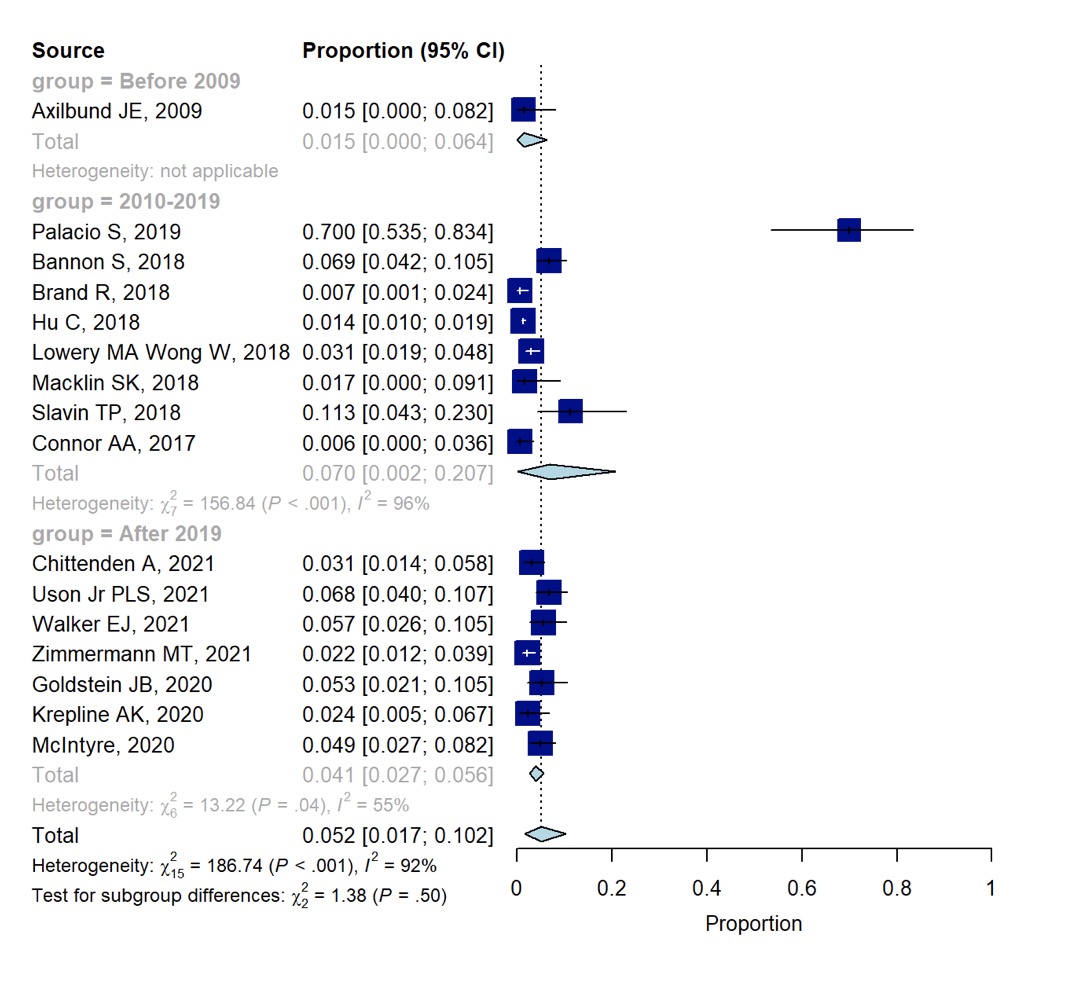


*4.2 Positive for any BRCA*

4.2.1 Funnel plot


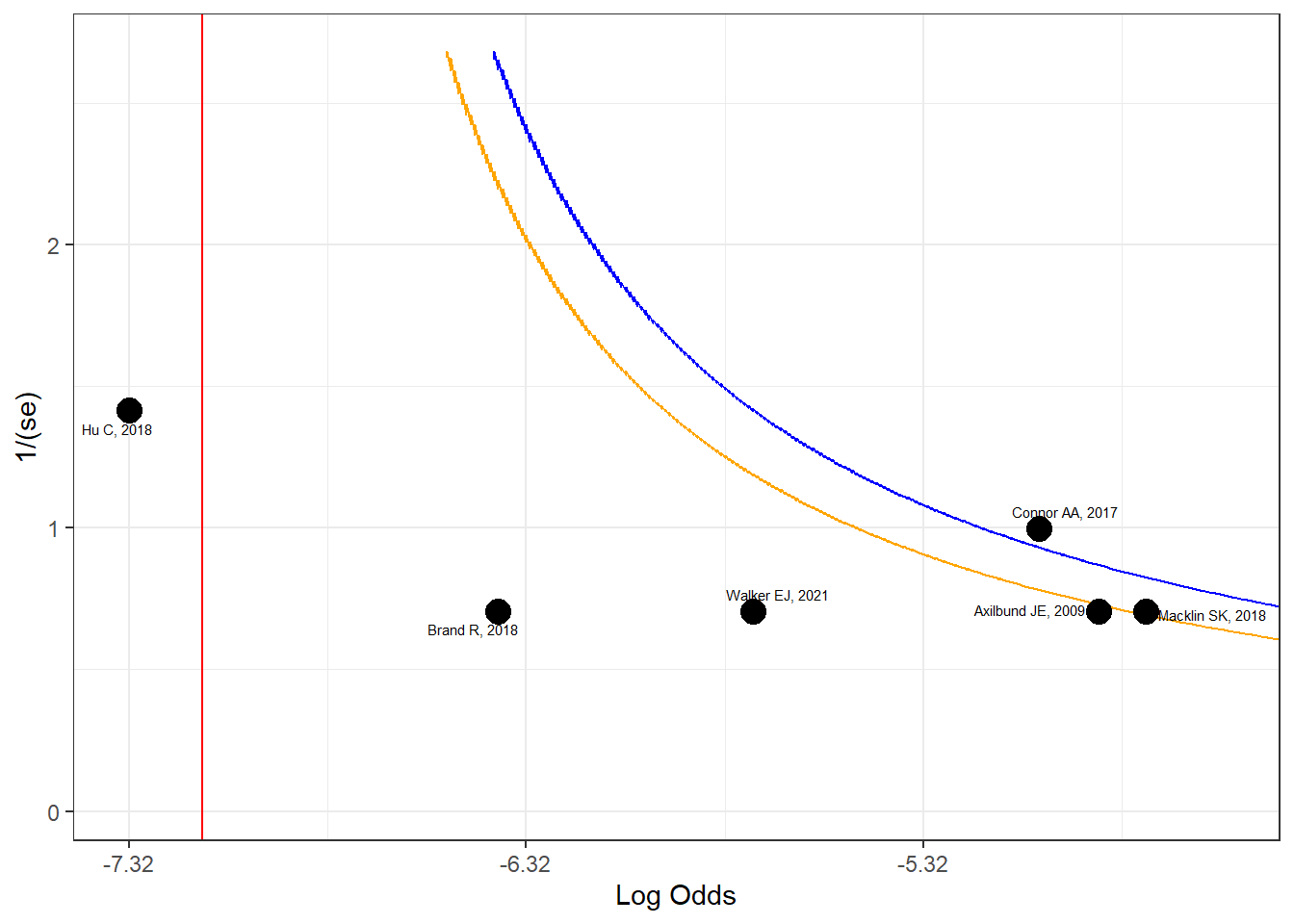


4.2.2 Metanalysis


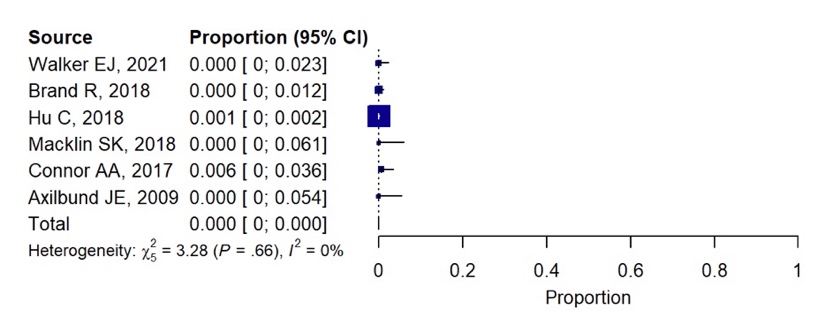


4.2.3 Temporal analysis


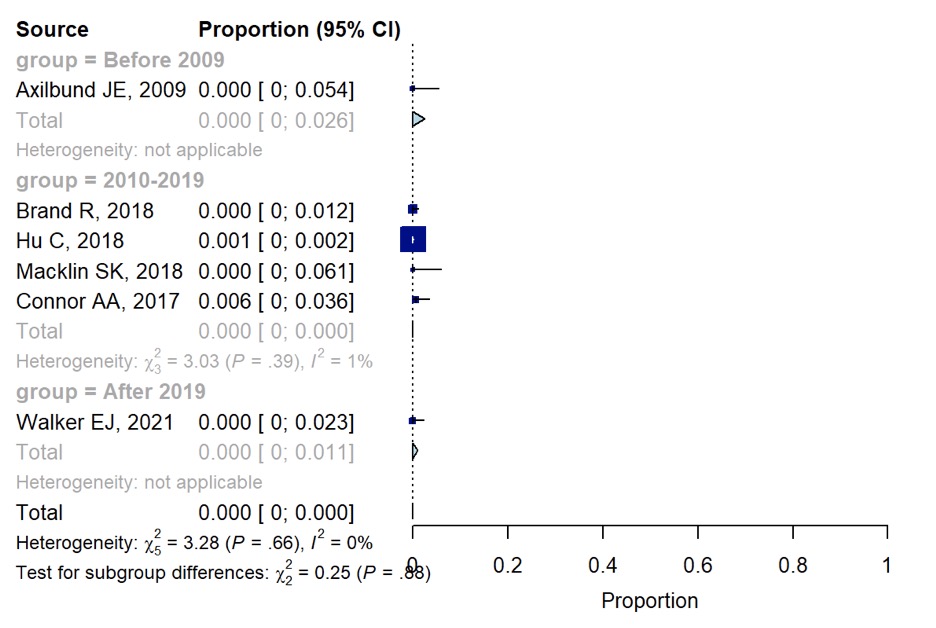

Supplement: Supplementaty Material 2 [file mmc2.docx]
