# Supplementary figures and images for: A systematic review and meta-analysis of germline BRCA mutations in pancreatic cancer patients identifies global and racial disparities in access to genetic testing
Source: ESMO Open. 2023 Feb 21;8(2):100881. doi: 10.1016/j.esmoop.2023.100881 (PMC10163165; doi:10.1016/j.esmoop.2023.100881)

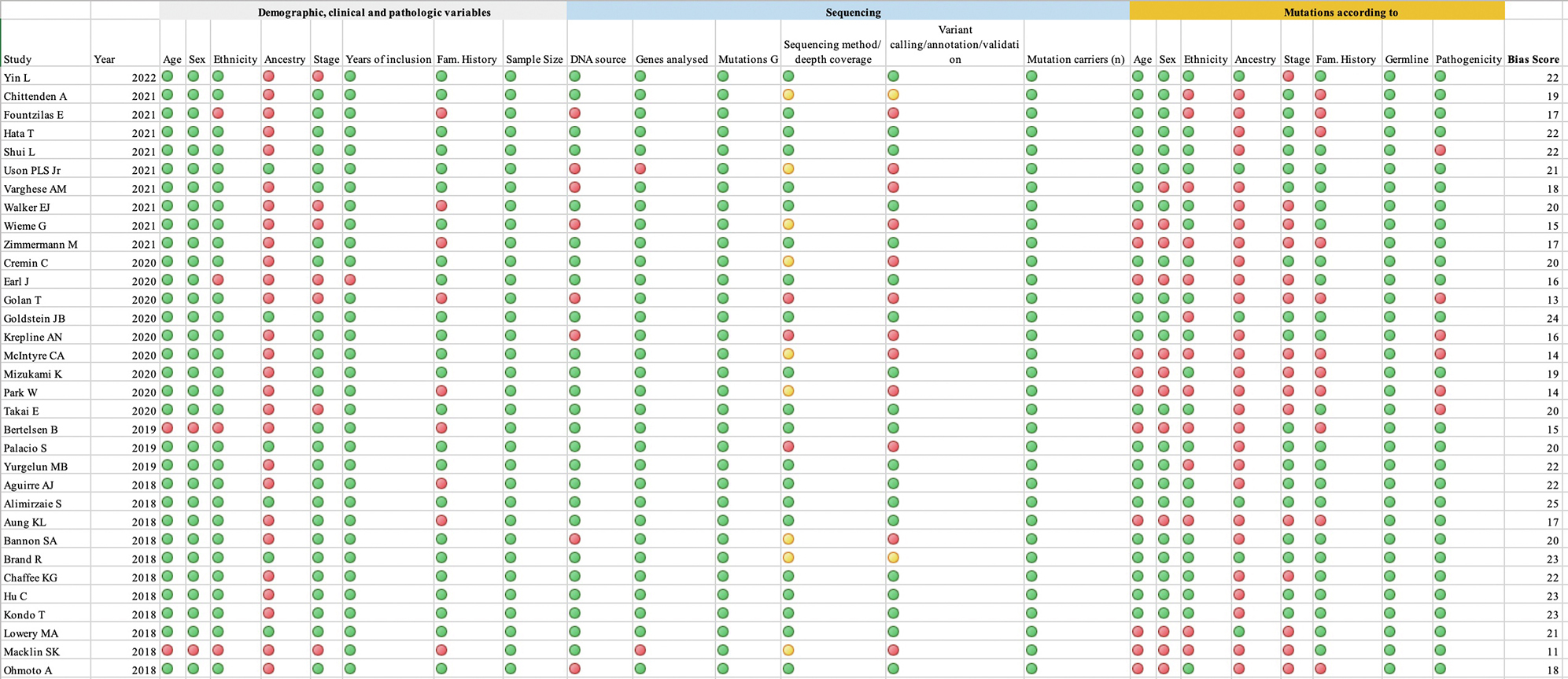

Supplement: Supplementaty Material 3 [file figs1.jpg]

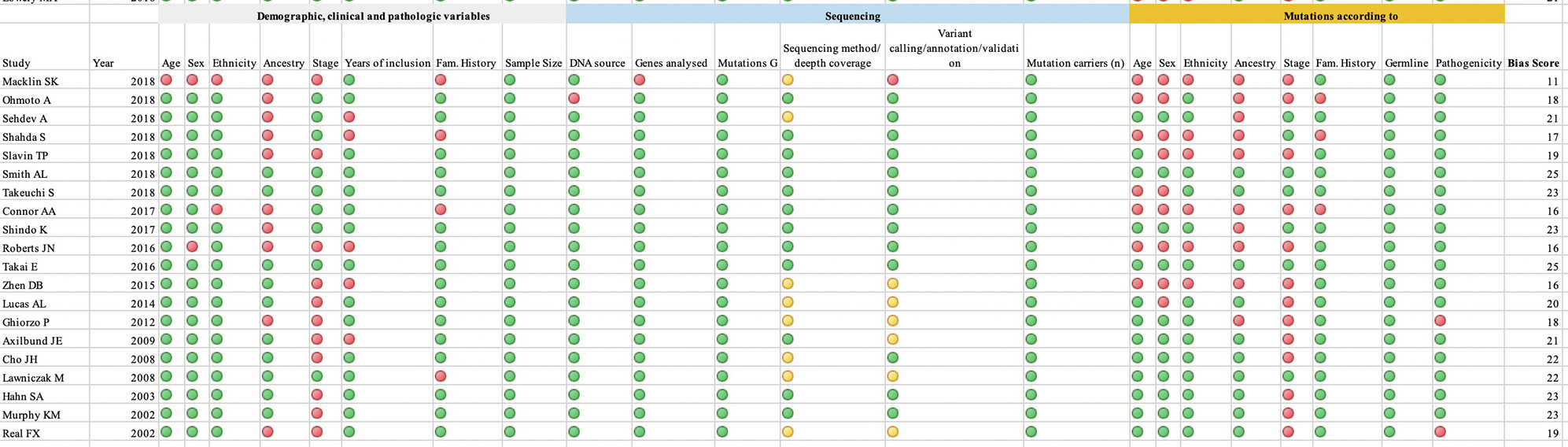

Supplement: Supplementaty Material 4 [file figs2.jpg]
